# Fast, multi-frequency, and quantitative nanomechanical mapping of live cells using the atomic force microscope

**DOI:** 10.1038/srep11692

**Published:** 2015-06-29

**Authors:** Alexander X. Cartagena-Rivera, Wen-Horng Wang, Robert L. Geahlen, Arvind Raman

**Affiliations:** 1 School of Mechanical Engineering, Purdue University, West Lafayette, Indiana, USA; 2 Birck Nanotechnology Center, Purdue University, West Lafayette, Indiana, USA; 3 Department of Medicinal Chemistry and Molecular Pharmacology, Purdue University, West Lafayette, Indiana, USA; 4 Purdue University Center for Cancer Research, Purdue University, West Lafayette, Indiana, USA

## Abstract

A longstanding goal in cellular mechanobiology has been to link dynamic biomolecular processes underpinning disease or morphogenesis to spatio-temporal changes in nanoscale mechanical properties such as viscoelasticity, surface tension, and adhesion. This requires the development of quantitative mechanical microscopy methods with high spatio-temporal resolution within a single cell. The Atomic Force Microscope (AFM) can map the heterogeneous mechanical properties of cells with high spatial resolution, however, the image acquisition time is 1–2 orders of magnitude longer than that required to study dynamic cellular processes. We present a technique that allows commercial AFM systems to map quantitatively the dynamically changing viscoelastic properties of live eukaryotic cells at widely separated frequencies over large areas (several 10’s of microns) with spatial resolution equal to amplitude-modulation (AM-AFM) and with image acquisition times (tens of seconds) approaching those of speckle fluorescence methods. This represents a ~20 fold improvement in nanomechanical imaging throughput compared to AM-AFM and is fully compatible with emerging high speed AFM systems. This method is used to study the spatio-temporal mechanical response of MDA-MB-231 breast carcinoma cells to the inhibition of Syk protein tyrosine kinase giving insight into the signaling pathways by which Syk negatively regulates motility of highly invasive cancer cells.

Mechano-chemical heterogeneity is a hallmark of living eukaryotic cells: the cell membrane is highly heterogeneous^1^,^2^, cell-cell and cell-extracellular matrix interactions are spatially localized through adhesion complexes^3^,^4^, cell motility requires asymmetric force generation^5^, subcellular organelles are discretely distributed within a cell^6^, and the cytoskeleton non-uniformly reinforces the cell’s rigidity^7^. These heterogeneities change dynamically with cell migration, morphogenesis, or by response to drugs^8^,^9^,^10^. Thus, there is a growing interest in technologies that are capable of mapping mechano-chemical heterogeneities within living cells with high spatio-temporal resolution.

Achieving high-speed mapping of nanomechanical properties of whole live eukaryotic cells (elastic modulus <100 kPa), over large areas (~50 × 50 μm^2^), and with significant range of topographies (cell height ~1–10 μm) has been a long standing challenge in AFM. This is due to the softness of live eukaryotic cells, which reduces the sensitivity of dynamic AFM observables such as amplitude and phase, and also because of the large height variations of live cells, which requires a high Z-piezo positioning range to track. Recent advances in AFM such as peak force tapping^11^,^12^ and multi-harmonic AFM^13^,^14^,^15^, have significantly improved material property mapping speeds on live cells compared to the conventional force-volume method. However, the acquisition time for a high resolution material property map over an entire eukaryotic cell remains >~10 minutes, which is insufficient for studying dynamic processes in cell biology^16^. Parallel developments in high speed scanning using high bandwidth electronics, scanners, and microcantilevers have imaged the topography of moderately stiff samples^17^,^18^,^19^,^20^,^21^ (elastic modulus >10 MPa) or the peripheral/flat areas of eukaryotic cells^22^,^23^ without mapping nanomechanical properties.

Here we present a method by which commercial AFM systems with directly excited cantilevers (magnetic, Lorentz force, or photothermal excitation) can be operated using a new cantilever deflection feedback scheme that boosts by at least one order of magnitude the speed of imaging whole live eukaryotic cells in solution as compared to AM-AFM. The method is fully compatible with emerging high speed AFM systems^17^,^18^,^19^,^20^,^21^,^22^,^23^,^24^,^25^. Recent advances in high speed AFM systems suggest that high speed scanning in specialized AFM systems using deflection feedback is possible^26^. Thus, the approach described in the present work should, in principle, be compatible with high speed AFM systems also. We conclude that; (a) the use of cantilever mean deflection as feedback signal instead of amplitude can boost by 1 order of magnitude the speed of nanomechanical mapping using resonant cantilevers over live cells in culture media. (b) The observables acquired from directly excited cantilevers scanning over cells with mean deflection feedback can be easily converted to quantitative local mechanical properties using advanced continuum mechanics models. (c) The method can be extended to multi-frequency approaches, for example by simultaneous excitation of the two fundamental eigenmodes of the cantilever and the observables can be used to map the viscoelastic response of cells at two widely space frequencies confirming that classical viscoelastic frequency dependence is present. (d) These advances permit, for the first time, the observation of nanomechanical spatio-temporal response of the cortical actin cytoskeleton including the formation and movement of lateral actin bands characteristic of the retrograde actin flow machinery rapidly formed by inhibiting Syk expression in MDA-MB-231 breast cancer cells.

Taken together, these findings suggest that the method allows the study of time varying heterogeneous physical properties of live cells during dynamic processes over large areas that was not heretofore possible.

## Results

### Dynamic AFM design for fast imaging of live cells

**Figure 1:**
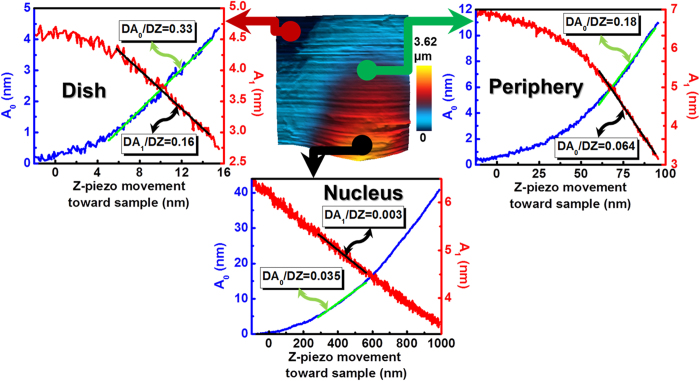
Choosing the right feedback signal for high-speed imaging of live cells. The Z sensitivity of *A*_0_ and *A*_1_ signals, 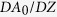 and 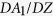, are measured on a fibroblast cell nucleus (black arrow), periphery (green), and gelatin-coated glass dish (red) in order to identify the most sensitive feedback channel while scanning a live cell.

Boosting the scanning speed of dynamic AFM on live cells requires the appropriate choice of feedback channel. We first tested the sensitivities *dA*_0_*/dZ* and *dA*_1_*/dZ* on a live cell of the cantilever mean deflection *A*_0_ and first harmonic amplitude *A*_1_ (the traditional feedback channel). A Lorentz force excited soft microcantilever (calibrated stiffness *k*_*cant*_ = 0.08 N/m, resonance frequency *ω*_*dr*_ = 8 kHz) is made to approach and press down on the surface of a live rat fibroblast cell in culture medium. As shown in Fig. 1, *dA*_0_*/dZ* is ~12, ~3, and ~2 times larger than *dA*_1_*/dZ* on the nuclear region, cell periphery, and the gelatin-coated dish, respectively. The low sensitivity of *A*_1_ on a live cell stems from two effects: (i) the small repulsive force gradients on a live cell which only perturb slightly the resonance frequency of the freely vibrating microcantilever, and (ii) the low Q factor which ensures that small force gradients do not change the amplitude appreciably. This finding is consistent with the work of Cartagena *et al.*^15^. This makes *A*_0_ the best choice for feedback in order to increase the imaging speed on soft eukaryotic cells, while *A*_1_ and the corresponding phase lag *ϕ*_1_ can be used as observables for quantifying local mechanical properties.

There are two important reasons why feedback on the *A*_0_ signal enables cell topography tracking at higher speed and stably when compared to AM-AFM; (a) First of all, the feedback loop gains used are 10–100 times larger when using amplitude feedback in AM-AFM on a live cell compared to *A*_0_ feedback. This is a direct consequence of stronger gradient of the *A*_0_ signal with respect to Z. In principle, the lack of sensitivity of amplitude to Z-piezo motion can be compensated by a larger feedback, but if the feedback gain is very high on the cell then the AFM becomes unstable when it scans the stiffer/peripheral parts of the cell and the substrate. (b) Secondly, for a given high speed scanning rate, tracking the large changes in height over a cell challenges the Z-piezo range of most available commercial AFM’s. With amplitude feedback, large changes in Z are needed to regulate the amplitude and can sometimes be at the limit of the Z-piezo positioning tracking range of the Z-piezo scanner. These trade-offs between scanning speed and scanning over large height changes is well-known and approaches using secondary actuators have been proposed recently^27^,^28^. However, here we focused on improving material property mapping speed on live cells stably using current commercial AFM systems.

**Figure 2:**
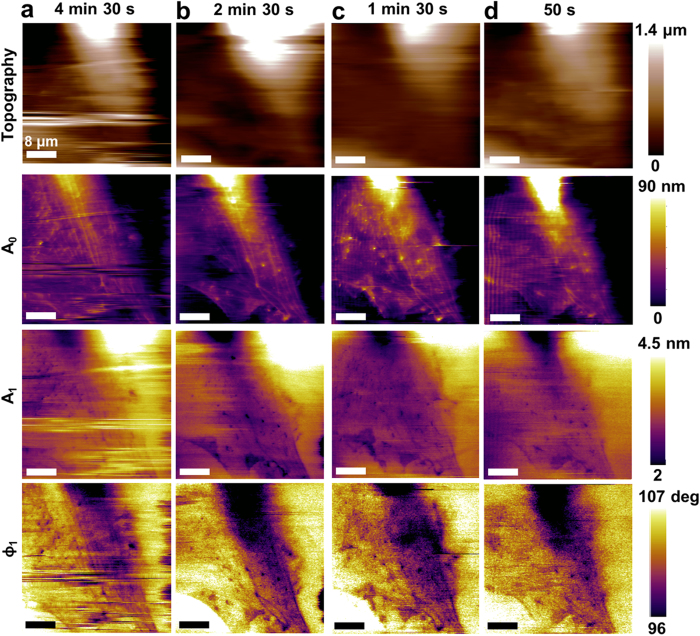
Influence of imaging speed on spatial resolution. Topography and observables maps (*A*_0_, *A*_1_, and *ϕ*_1_) are acquired using *A*_0_ feedback at different scan speeds: (**a**) 4 min 30 s, (**b**) 2 min 30 s, (**c**) 1 min 30 s, and (**d**) 50 s that clearly show improved scanning speed compared to AM-AFM. AM-AFM image of the same area of cell takes roughly 15 minutes. The observables maps show cell heterogeneities and the method does not permanently damage the cell. However, at high scanning speeds ripples begin to appear in the image due to the limited bandwidth of the Z piezo used in commercial AFM systems. Imaging parameters; 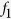 = 8.01 kHz, *k*_*cant,1*_ = 69.5 pN/nm, Q = 1.6, and 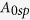 = 18 nm. The scale bar on images represents 8 μm (size; 40 × 40 μm^2^, pixels; 256 × 256).

In order to image, the resonant microcantilever is made to approach a live cell until a setpoint deflection *A*_0_ of ~10–20 nm is reached, corresponding to a force *k*_*cant*_ *A*_0_ of ~800–1600 pN. Even at such small forces the microcantilever does not intermittently tap the cell but rather oscillates while in permanent contact with the nuclear and peripheral regions of live fibroblast cells. Using *A*_0_ feedback, successive topography images were obtained (Fig. 2) on a live rat fibroblast in culture medium varying the scan frequency from 1 Hz to 6 Hz at 256 point per line. The microcantilever remains in permanent contact with the cell surface during these scans even while *A*_0_, and thus the average force applied, changes due to the large changes in topography on the cell. Simultaneous maps of *A*_0_, *A*_1_, and *ϕ*_1_ in Fig. 2 are related to local mechanical properties of the cell membrane, cytoskeleton, and other subcellular components^10^. The fastest acquisition time for the images was 50 s, which is 20 times faster than previously reported AM-AFM images on whole live eukaryotic cells^13^,^15^. More than 7680 pixels of local mechanical property information can be acquired every 10 seconds, bringing the time-resolution of AFM based material property mapping closer to that of speckle fluorescence microscopy which is used to image cytoskeletal dynamics.

Note that mean force feedback is not new in off-resonance AFM methods^29^,^30^,^31^,^32^. However, mean force feedback is rarely used in resonant AFM techniques. The use of this feedback while imaging live cells in culture media with resonant cantilevers allows a significant increase in scanning speed for nanomechanical mapping compared to traditional amplitude feedback.

### Theory for measuring quantitative nanomechanical properties

**Figure 3:**
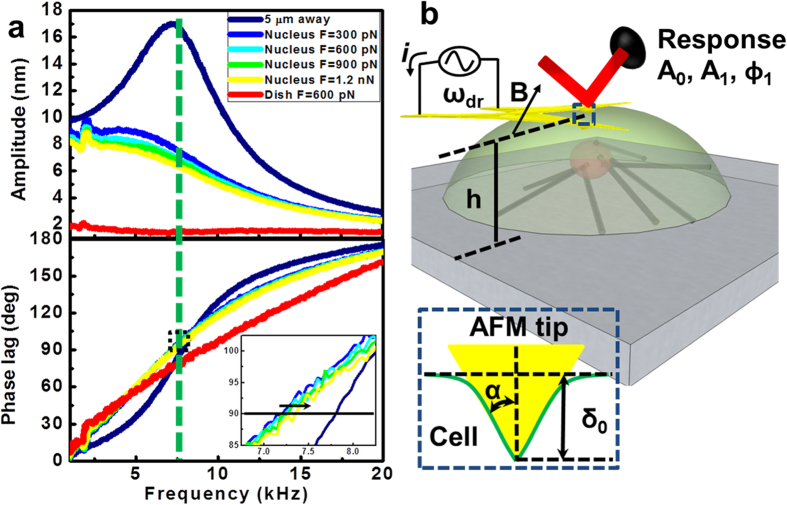
Quantification of local nanomechanical properties from AFM observables. (**a**) TR400 cantilever vibration transfer functions acquired off the sample, in contact with the nucleus increasing the applied force and in contact with the dish. (**b**) Schematic of a cell with a Lorentz force excited cantilever driven at resonant frequency ω_*dr*_ and interacting in full contact with the cell. Inset is a section model of the pyramidal AFM tip with half-opening angle *α*, and indentation *δ* indenting an elastic half-space.

Two important facts facilitate the conversion of the observables to quantitative local mechanical properties. First, as can be observed in Fig. 3a, the Lorentz force excited microcantilever suffers only a small change in resonance frequency when pressed against the cell compared to when it is far from the cell. This is observed by the small change in natural frequency in the phase shifts plots ~250 Hz from far to in contact with the cell surface. Moreover, the effective Q factor of the microcantilever decreases to <1 when in contact with the cell. Secondly, the microcantilever oscillation amplitude *A*_1_ (1–5 nm) is usually much smaller than the mean indentation *δ*_0_(10–100 nm) into the live cell Thus, Lorentz excited microcantilever in contact with a live cell can be well described by classical, well-behaved transfer functions that relate the observables *A*_1_, *ϕ*_1_, the calibrated cantilever spring constant *k*_*cant*_, the Q factor, and peak resonance frequency to a linearized spring and dashpot 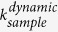, 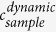
of a classic two-element Kelvin-Voigt viscoelastic model^33^ that represents the local nanomechanical properties (see Supplementary Information Section B).

Local viscoelastic constitutive parameters such as storage and loss modulus can also be derived from such maps. For example, using the Sneddon’s conical tip model, which is frequently used for cell mechanics, simple analytical formulas can be derived that relate the local elastic storage and loss viscous moduli 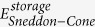, 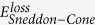, and mean indentation *δ*_0_ to the acquired observable maps *A*_0_, *A*_1_, and *ϕ*_1_ (see Supplementary Information Section C). However, Sneddon’s conical tip model assumes indentation into half-space and thus is applicable for moderate indentations on thick parts of cells^34^. More recently, a correction to this model has been proposed, the Bottom Effect Cone Corrected (BECC) model^35^, which systematically takes into account the finite thickness *h* of the soft sample. Both these models assume non-adhesive contact of an axi-symmetric tip with a linearly elastic sample. When the indentation stresses become large, hyperplastic indentation theories like^36^ can be used. In principle, all such approximate analytical theories can be related to the local elastic storage modulus 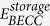 at a mean indentation *δ*_0_ and thus extracted using this technique. We have developed an algorithm that reads *h* from the topography image and the corresponding 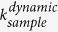, and 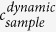 maps and computes those values of 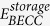, 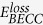, and 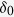 that best fit the BECC model with a viscoelastic extension (see Supplementary Information Section D). This method is expected to lead to better estimates of viscoelastic moduli on thinner parts of the sample, however, converting the observables into local property maps is more difficult since it requires a nonlinear least squares fit algorithm. This approach to convert the observables into constitutive parameters can, in principle, be extended to other cell mechanics models^37^,^38^ so long as the number of unknown constitutive parameters is less than the number of observables.

### Fast quantitative nanomechanical properties maps of fibroblasts cells

**Figure 4:**
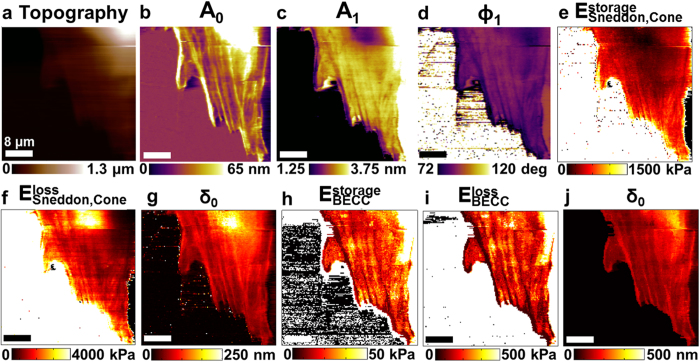
Mapping nanomechanical properties of live rat fibroblast cells. (**a**) Topography image of a live rat fibroblast cell scanned using a Lorentz force excited microcantilever with *A*_0_ regulation (see Materials and Methods). (**b–d**) Multi-harmonic images of (*A*_0_, *A*_2_, *ϕ*_1_) acquired simultaneously with topography showing high resolution subcellular contrast related to the local physical properties. (**e–g**) Maps of local elastic storage modulus 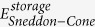 and viscous loss modulus 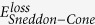, and mean indentation *δ*_*0*_ extracted from the multi-harmonic data and using the viscoelastic Sneddon’s model described in the Supplementary Information Section C. (**h–j**) Maps of local storage modulus 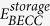, local loss modulus 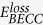, and mean indentation *δ*_*0*_ extracted using the multi-harmonic data and the viscoelastic BECC model described in the Supplementary Information Section D. Imaging parameters; 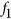 = 7.9 kHz, *k*_*cant *_= 87.41 pN/nm, *Q* = 1.75, α = 35°, and 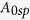 = 24 nm. The scale bar on images represents 8 μm (size; 40 × 40 μm^2^, pixels; 256 × 256, acquisition time; 4 min 30 s).

We compare these methods in Fig. 4 using observable maps of rat fibroblast cells in culture medium (see Materials and Methods). Fine detail of subsurface features such as actin filament bundles (stress fibers) and the nuclear complex are clearly displayed in the physical property maps. The 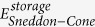 map shows that the regions with actin bundles are stiffer compared to those where they are not present. This implies that the tip presses sufficiently into the cell to detect the increased stiffness due to the actin bundles^15^. As expected, the 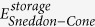 map also demonstrates a higher Young’s modulus in the thinner/peripheral region compared to the thicker nuclear/perinuclear area. This thickness dependence is remarkably absent in the 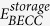 map, since the BECC model accounts for the finite thickness of the sample. On the other hand, the use of the BECC model in this method requires the use of topography (height) information. Any noise or smear in the topography will also be reflected in the extracted property maps using the BECC model. Generally speaking, the elastic modulus values measured on these cells using dynamic AFM methods are in line with previous measurements^10^,^13^,^15^,^39^,^40^,^41^ and are 3–5 larger than those measured using quasi-static AFM methods due to differences in drive frequency and indentation^15^. The validation for the use of dynamic AFM observables of Lorentz excited cantilevers for viscoelastic properties of living cells has been recently presented^15^. However, here we described how to acquire the same observables and the mapping of nanomechanical properties at much higher scanning speed.

### True viscoelastic property mapping using two widely spaced frequencies

**Figure 5:**
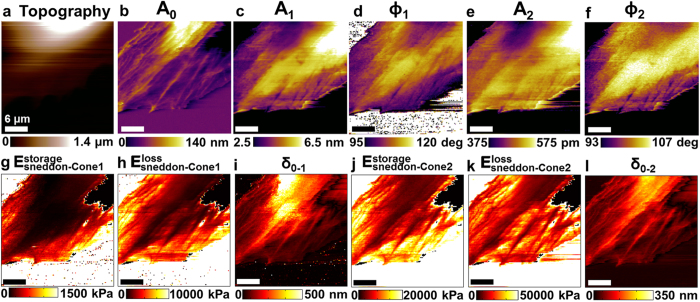
Application to bimodal AFM operation. Multi-frequency observables images (**b**) mean deflection *A*_0_, first and second flexural eigenmodes amplitudes and phases (**c–d**) *A*_1_, *ϕ*_1_, and (**e–f**) *A*_2_, ϕ_2_ obtained simultaneously using the previously described mean deflection regulation. (**g-i**) Maps of local elastic storage modulus 
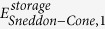
, viscous loss modulus 
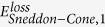
, and indentation *δ*_0–1_ extracted from the measured first mode data (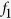 = 7.06 kHz) using the theory described in the text and Supplementary Information Section E. (**j–l**) Maps of local elastic storage modulus 
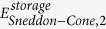
, viscous loss modulus 
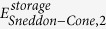
, and indentation *δ*_0–2_ extracted from the measured second mode data (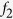 = 61.33 kHz) using the theory described in the text and Supplementary Information Section E. This shows multi-frequency can be combined with this technique enabling additional compositional contrast channels revealing unrelated subcellular features. Topography and multi-modal observables were not taken simultaneously. Imaging parameters; *k*_*cant,1 *_= 77.19 pN/nm, 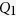 = 1.7, *k*_*cant,2*_ = 1.33 N/m, 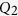 = 3, and 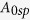 = 36  nm. The scale bar on images represents 6 μm (size; 30 × 30 μm^2^, pixels; 256 × 256, acquisition time; 2 mins 30 s).

The proposed method also allows for viscoelastic property mapping at widely spaced frequencies by simultaneously exciting the cantilever in the fundamental and second flexural eigenmode. Multi-modal excitation such as bimodal^42^,^43^,^44^,^45^ or trimodal^46^ are able to boost the number of compositional contrast channels but have not been demonstrated for fast scanning on live cells. Figure 5 shows the application of this high-speed quantitative nanomechanical mapping with bimodal excitation where observables now become *A*_0_, *A*_1_, *ϕ*_1_, *A*_2_, and *ϕ*_2_ representing the mean deflection, 1^st^ eigenmode amplitude and phase and 2^nd^ eigenmode amplitude and phase, respectively. The maps of *A*_0_, *A*_1_, *A*_2_ and *ϕ*_1_, *ϕ*_2_ were acquired for a live fibroblast cell in culture media (Fig. 5 (a–e)). Using the first and second eigenmode observables acquired in a single scan of fibroblast cells, we extracted the maps of 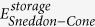 and 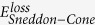 at 7 kHz and 61 kHz (see Supplementary Information Section C for details). Classic viscoelastic frequency dependence is observed, where there is an evident increase in the cell stiffness and damping of ~10–50 and ~1–3 times, respectively at 61 kHz compared to at 7 kHz. This is in line with prior measured viscoelastic properties of hydrogels and live fibroblast cells^15^,^47^. Furthermore, the viscoelastic loss tangent tan*δ* when the measurement frequency increases from 7 kHz to 61 kHz (see Supplementary Information Section F), is reduced by ~0.3–0.9 in line with prior measurements on hydrogels^47^. Such high frequency viscoelastic measurements on live cells are particularly relevant for cell mechanobiology, since existing power-law based microrheological models^48^ of live cells are usually only valid for small frequencies (<1 kHz).

### Spatio-temporal nanomechanical response of MDA-MB-231 breast carcinoma cells to the inhibition of Syk protein tyrosine kinase

**Figure 6:**
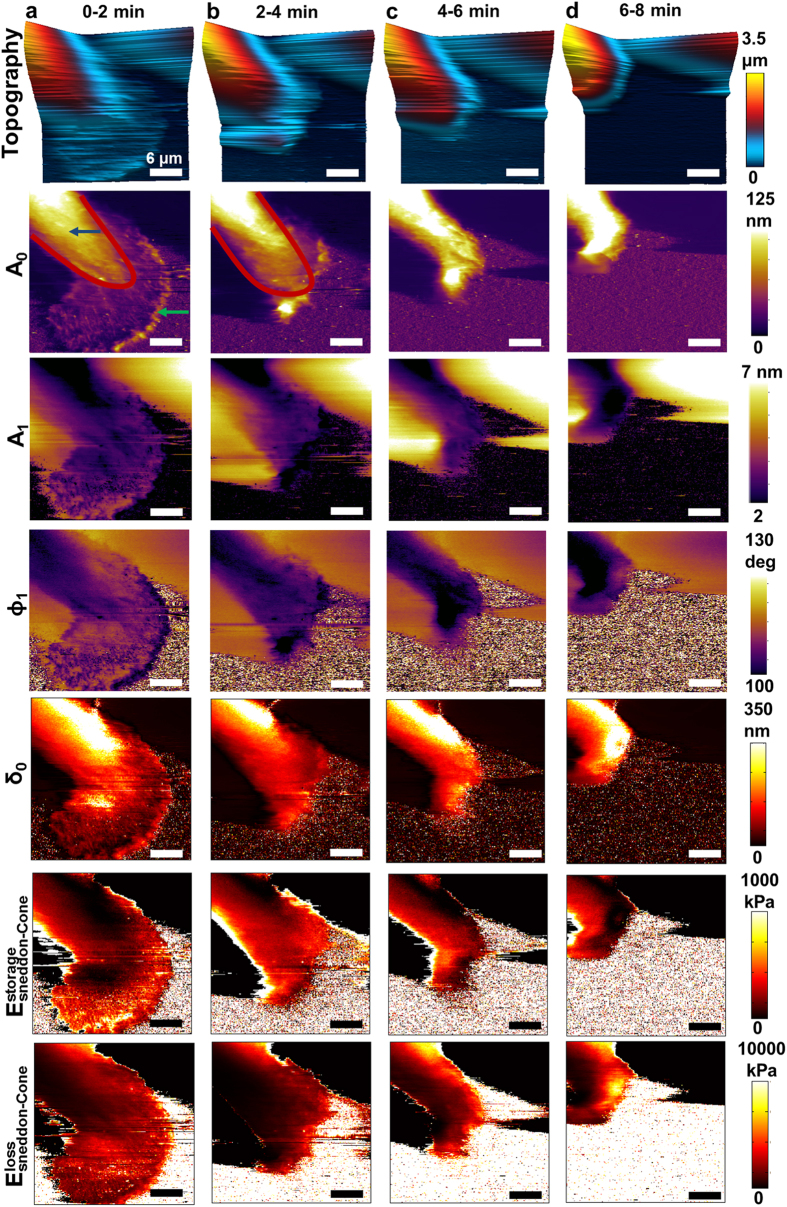
Application to cancer cell mechanobiology; tracking the fast time-varying changes in nanomechanical heterogeneities of MDA-MB-231 breast cancer cells upon inhibition of Syk-AQL-EGFP protein tyrosine kinase with 1-NM-PP1. (**a**) Topography, observables, and nanomechanical property images using the Sneddon’s contact mechanics model before adding the inhibitor. (**b–d**) Time-series of images taken at intervals of 2 minutes each after adding the inhibitor. Fast changes in morphology and nanomechanical property maps are observed. Imaging parameters; 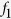 = 7.1 kHz, *k*_*cant,1*_ = 74.04 pN/nm, 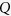 = 1.67, 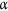 = 35°, and 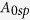 = 20 nm. The scale bar on images represents 6 μm (size; 30 × 30 μm^2^, pixels; 256 × 256, acquisition time; 2 min).

The increased spatio-temporal resolution of this method enables the study of time-varying nanomechanical heterogeneities in human cells. For this study, we examined MDA-MB-231 human breast cancer cells expressing Syk, a tumor suppressing kinase. Recently, it has been shown that Syk expression in MDA-MB-231 cells modifies their mechanical phenotype^49^, however, the kinetics of this effect are not known. To assess this, MDA-MB-231 cells were generated that express a fluorescently tagged analog-sensitive Syk mutant (Syk-AQL-EGFP) engineered to be uniquely sensitive to an orthogonal inhibitor^50^. Syk when expressed in highly invasive breast cancer cells, acts as a tumor suppresor^51^,^52^ by inhibiting cell motility^53^ and enhancing cell-extracellular matrix interactions. However, it is not known if this is due to Syk-induced changes in gene expression^54^ or to rapid alterations in protein phosphorylation. In this study, the activity of Syk was selectively inhibited by the addition of 1-NM-PP1, which binds selectively to Syk-AQL-EGFP to rapidly block its activity to allow the cells to regain their initial highly motile properties^50^. This approach was chosen to avoid potential off-target effects of kinase inhibitors and is illustrated by the observation that cell adhesion is attenuated by 1-NM-PP1 in cells expressing Syk-AQL-EGFP, but not in cells expressing Syk-EGFP (Supplementary Information Section G). The inhibition process allows the transition between a tumor suppressed state to a fully malignant state to occur in ~10 minutes, making it an ideal model system for fast nanomechanical property imaging. Figure 6 (a–d) shows a sequence of topography, observables and nanomechanical property maps acquired at two minute intervals of a MDA-MB-231 cell immediately following the inhibition of Syk activity. Using the obtained observables, we extract the local nanomechanical properties of the time series of images using the theory described in the Supplementary Information Section C. Since these cells are much softer than the fibroblasts and are also more motile, their topography is not as well defined over the course of imaging as for live fibroblasts, thus we map the 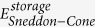, 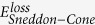, and *δ*_0_.

Time-varying changes in the cell periphery were observed that included a rapid retraction of the leading edge and disassembly of focal adhesions. Figure 6 column (a) shows two distinct regions, the voluminous perinuclear region (blue arrow), and the flat leading edge (green arrow). The two regions have different moduli 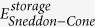, 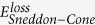, with the perinuclear region being stiffer and less viscous than the periphery. After adding the inhibitor, comparing Fig. 6b with Fig. 6a, there is an evident contraction of the leading edge accompanied by a rapid decrease in focal adhesions. Moreover, there is an increase in area of the high modulus region of ~30% (red line). Figure 6 (c and d), however, show a progressive decrease in the voluminous region due to a subsequent loss of focal adhesions combined further with disassembly of the cytoskeletal network.

### Syk modulates cortical actin cytoskeleton

The cell periphery had largely contracted within 2 minutes of addition of the inhibitor. To explore further the kinetics of these changes, we monitored MDA-MB-231 cells at 90 second intervals following addition of the Syk-AQL-EGFP inhibitor (see Supplementary Information Section H, movies S1–S6). Interestingly, in the AFM multi-harmonic observable movies S1–S3, rapid changes in the cytoskeletal architecture at the cell periphery could be visualized within 1.5 min including the formation and movement of lateral actin bands or transverse arcs characteristic of retrograde actin flow that preceded the release of focal adhesions^55^. Thus, the rapid loss of Syk activity was correlated with dramatic rearrangements in the cortical actin cytoskeleton, consistent with reports of Syk modulating cortical actin dynamics in B cells and platelets^56^,^57^. Movies S3–S6 show time-varying changes in the nanomechanical properties due to the dramatic rearrangement of the lamellipodial cortical actin cytoskeleton following the addition of the Syk-AQL-EGFP inhibitor. Thus, the substantial changes in the cellular architecture resulting from the expression of Syk are rapidly reversed upon its inhibition, indicating that Syk modulates cytoskeletal dynamics via reversible phosphorylation. We also have demonstrated a role for Syk in modulating microtubule dynamics by stabilizing microtubule polymers^49^, which we propose contributes to the dramatic changes in the internal structure and nanomechanical properties of the cell.

## Discussion

Here we have demonstrated a new fast scanning quantitative dynamic AFM method for nanomechanical imaging of heterogeneous live cells in solution by using the cantilever mean deflection as feedback signal instead of standard amplitude reduction. This new method was able to achieve a 10–20-fold improvement in imaging throughput compared to AM-AFM. Quantitative nanomechanical maps on live eukaryotic cells are acquired with a high-throughput of around ~7680 pixels every 10 seconds, closing the barrier between AFM imaging material property speeds and cellular process dynamics. The use of advanced continuum contact mechanics models (e.g., BECC with viscoelasticity) facilitated the conversion of dynamic AFM observables to quantitative constitutive property maps. The method can also be easily combined with existing bimodal or trimodal techniques for simultaneously mapping viscoelastic properties and widely spaced frequencies (7 and 61 kHz). The increased temporal resolution of this method enables the study of dynamic changes in cellular properties that accompany the inhibition of a tumor suppressor that, in MDA-MB-231 breast cancer cells, converts them from fully malignant to nonmetastatic^50^. The method is fully compatible with emerging high speed scanning AFM systems. The advances described herein, when coupled with other hardware improvements, will enable studies of near real time changes in the morphology and subcellular nanomechanical properties of live cells that are relevant to understanding fundamental cellular biophysical dynamics, the actions of new drugs, and tumor metastasis.

## Materials and Methods

### Cell Culture

Rat fibroblast cells were cultured in Dulbecco’s Modified Eagle’s Medium (DMEM) (Invitrogen) containing D-glucose (1000 mg l^−1^), 10% fetal bovine serum (FBS) (Invitrogen), 1% penincillin-streptomycin (Invitrogen), and 0.1% amphotericin B (Sigma Aldrich). The human breast cancer cell line, MDA-MB-231, was obtained from ATCC and cultured in DMEM containing 10% FBS, 100 U ml^−1^ penicillin, and 100 μg ml^−1^ streptomycin. Cells initially grown on plastic flasks were trypsinized with 0.5% trypsin/EDTA solution and the cell suspension was deposited on the bottom glass of a fluorodish (World Precision Instruments) pre-coated with 0.1% gelatin in water (StemCell Tech.). The cells were grown on the dish 1–2 days prior to imaging and maintained in an incubator at 37 °C in a 5% CO_2_ atmosphere to ensure complete spreading. Before AFM measurements, the cells were rinsed twice with fresh PBS and fresh culture media was added. All measurements were performed in culture media at 37 °C by placing the dish on a heating stage. For studies on the inhibition of Syk protein tumor suppressor activity, MDA-MB-231 breast cancer cells expressing Syk-AQL-EGFP were constructed using the Lenti-X Tet-On Advanced Inducible Expression System (Clontech). MDA-MB-231 cells were first generated to constitutively express the tetracycline-controlled transactivator, rtTA. These cells were then infected with lentiviral particles packaged with pLVX-Tight-Puro-Syk-AQL-EGFP. Syk-AQL-EGFP was generated by mutations of three amino acids (R428Q/M429L/M442A) within the Syk kinase domain (see ref. 50 materials and methods). After 48 h, cells were selected with 1 μg/ml puromycin and screened for expression by Western blotting and visualized by fluorescence microscopy. Syk-AQL-EGFP was induced by the addition of 1 μg/ml of doxycycline (Sigma Aldrich) and incubated for ~24 h for full expression of Syk-AQL-EGFP within individual cells. After finding a green cell for inhibition experiments, the inhibition of Syk was performed by the addition of 5 μM 1-NM-PP1 (Cayman Chemical Co.), which will bind selectively to Syk-AQL. Immediately after adding the inhibitor high-speed imaging was resumed to study the changes.

### AFM imaging

We used the MFP-3D-Bio AFM system (Asylum Research) mounted on an IX-71 Olympus inverted optical microscope for easy positioning of cells. The AFM has a heating stage that keeps the sample temperature constant at 37 °C. Soft TR400PB microcantilevers (BL-TR400PB Olympus/Asylum Research) with nominal spring constant of 0.09 N/m, nominal tip radius of 42 nm (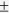
12 nm), and half-opening angle *α* = 35° were employed. The cantilever is mounted on the iDrive Lorentz force excitation holder that enables direct excitation of the cantilever by passing an alternating current trough the V-shape cantilever creating an electric field that interacts with a magnetic field created by a permanent magnet to create a net oscillating force on the cantilever^58^. After the laser was properly aligned on the cantilever, a force–distance curve was performed on an infinitely stiff substrate (mica) in the same media solution as the cell imaging to calculate the deflection sensitivity of the cantilever. The cantilevers spring constant was calibrated by the thermal noise fluctuations method^59^.

We switched the feedback from conventional resonant frequency oscillation amplitude *A*_1_ to cantilever mean deflection *A*_0_. This was done by using the piezoresponse force microscopy mode, allowing the increase in scanning speed from 0.25 Hz to 6 Hz per line on whole live eukaryotic cells in media solution over areas of 30–70 μm by 30–70 μm at 256 by 256 pixels. The AFM cantilever was directly driven (Lorentz force excitation) at its first frequency flexural mode (6–8 kHz) when far from the sample and then engaged into the live cells. The amplitude far from the sample was selected to be approximately 8–18 nm. The deflection setpoint for imaging was ~20–35 nm (force ~1.5–2.5 nN) based on the mean deflection signal of the cantilever.

For bimodal imaging, both the first and second eigenmodes of the cantilever were simultaneously excited using Lorentz excitation. The second eigenmode amplitude far from the sample was chosen to be in the order of magnitude of that of the first mode free amplitude (~3–6 nm). The deflection setpoint for imaging was ~20–35 nm (force ~ 1.5–2.5 nN) based on the mean deflection signal of the cantilever. The equivalent spring constant of the second eigenmode was estimated by *k*_*cant,2*_ = 17.2*k*_*cant,1*_^60^.

Phase lag correction plays an important role for properly quantification of the nanomechanical properties of live cells in liquids. For the first mode of operation, initially we set the phase lag to be +90° when the drive frequency is tuned to resonance far from the sample substrate when no interaction forces were present. Because we used low Q factor cantilevers in liquids, the hydrodynamic squeeze film and added mass effects^61^,^62^ changed the phase at resonance far from the sample instead of +90° to ~74° (Q = 2)^15^. To correct for this phase shift, we subtract 14–16° to the obtained phase images. However, an important consideration for dual mode imaging is the initial phase assigned to the second mode resonance peak when no interaction forces are present. Because in the case of full contact with the soft sample in liquids the second eigenmode can be treated as a separate independent simple harmonic oscillator, we set the second phase lag to +90°. Then we scanned a sample and recorded the topography simultaneously with the material contrast channels *A*_0_, *A*_1_, *ϕ*_1_, *A*_2_, and *ϕ*_2_.

### Data Analysis

The acquired AFM images were 3D-rendered (as for topography data Fig.1) or the colormap modified for better appreciation of cells details and features in IGOR Pro 6.2 software (WaveMetrics). All computations and data processing were performed offline using MATLAB software (The MathWorks). For the data obtained from the new contact-resonance AFM mode, each set of multi-harmonic observable (*A*_0_, *A*_1_, *ϕ*_1_) or multi-mode (*A*_0_, *A*_1_, *A*_2_, *ϕ*_1_, and *ϕ*_2_) maps were synthesized using the mathematical relations described in the Supplementary Information to extract the constitutive viscoelastic parameters using Sneddon’s model *δ*_*0*_, 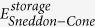 and 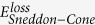 by directly solving the analytical formulas or the constitutive parameters using the BECC model *δ*_*0*_, 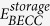 and 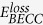 using a nonlinear least-squared fit algorithm to best fit the unknown properties to the dynamic AFM observables. For nonlinear least-squared approach the goodness of the fit was always tested by extracting residuals and resnorms with values around 0.1, confirming the applicability of the contact mechanics model to experimental data.

## Additional Information

**How to cite this article**: Cartagena-Rivera, A. X. *et al.* Fast, multi-frequency, and quantitative nanomechanical mapping of live cells using the atomic force microscope. *Sci. Rep.* **5**, 11692; doi: 10.1038/srep11692 (2015).

## Supplementary Material

Supplementary Information

Supplementary Video S1

Supplementary Video S2

Supplementary Video S3

Supplementary Video S4

Supplementary Video S5

Supplementary Video S6
