# Supplementary material for: Fast, multi-frequency, and quantitative nanomechanical mapping of live cells using the atomic force microscope
Source: Sci Rep. 2015 Jun 29;5:11692. doi: 10.1038/srep11692 (PMC4484408; doi:10.1038/srep11692)
Supplement: Supplementary Information [file srep11692-s1.pdf]

Supplementary Information

Fast, multi-frequency, and quantitative nanomechanical imaging of live cells using the atomic force microscope

Alexander X. Cartagena-Rivera[1,2,±], Wen-Horng Wang[3], Robert L. Geahlen[3,4] and Arvind Raman[1,2,*]

[1]School of Mechanical Engineering, Purdue University, West Lafayette, Indiana, USA
[2]Birck Nanotechnology Center, Purdue University, West Lafayette, Indiana, USA
[3]Department of Medicinal Chemistry and Molecular Pharmacology, Purdue University, West Lafayette, Indiana, USA
[4]Purdue University Center for Cancer Research, Purdue University, West Lafayette, Indiana, USA
±Present address: Laboratory of Cellular Biology, Section on Auditory Mechanics, National Institute on Deafness and Other Communications Disorders, National Institutes of Health, Bethesda, Maryland, USA

This supplementary documentation has been created to provide additional information to support the main text. The sections discussed in this document are:

a. **Direct versus other microcantilever excitation methods in tip-surface coupled AFM in liquids**
b. **Linearized spring and dashpot of a two-element Kelvin-Voigt viscoelasticity model**
c. **Viscoelastic Sneddon's contact mechanics model**
d. **Bottom effect cone correction (BECC) of the Sneddon's model with viscoelasticity extension**
e. **Bimodal AFM imaging for viscoelastic property mapping**
f. **Additional images of nanomechanial properties of rat fibroblast cells**
g. **Adhesion experiments**
h. **How well the BECC contact mechanics model fits the experimental data**
i. **Movies of additional MDA-MB-231 human breast cancer cell expressing Syk and treated with inhibitor**

a. Direct versus other microcantilever excitation methods in tip-surface coupled AFM in liquids

Many directly and indirect excitations are commonly used for dynamic atomic force microscopy in liquids (1-5). However, for extracting quantitative information direct excitation is required since it leads to well-defined microcantilever dynamics in liquids. The most frequently used direct excitation methods are magnetic (1-2), and

photothermal (3-4) actuation. Indirect excitations are acoustic (1) and sample (5) actuation. Below is a brief description of each excitation method:

- Acoustic mode is the most widely used method and consists of a piezoelectric transducer or dither piezo attached to the cantilever holder and vibrated at high frequencies to excite the microcantilever. This excitation method not only drives the cantilever but also the chip, holder and surrounding liquid. This generates an effect called forest of peaks that masks out the real microcantilever vibration response. A second, more difficult effect to understand, is that of fluid borne excitation which has a major influence on cantilever dynamics even if the forest of peaks effect is resolved (6).
- Sample excitation can be done with a piezo-electric transducer located underneath the sample and vibrated. The microcantilever is then brought into direct physical contact with the vibrating sample exciting the cantilever. An issue is that this excitation excites the sample and the liquid creating unwanted resonances that masks out the real microcantilever vibration response.
- Photothermal excitation uses a high powered laser to excite AFM microcantilevers in liquids yielding clean/smooth resonant peaks without spurious peaks and with a wide frequency bandwidth. However, phototermal efficiency is low requiring large amounts of laser power to mechanically actuate the cantilever a few nanometers, resulting in local heat that can potentially damage sensitive samples and accelerate liquid evaporation.
- For direct “magnetic” excitation, as the name implies, only the microcantilever is directly excited and a clean vibration response is obtained. There are two ways of doing this: (a) magnetic, which consists of a paramagnetic coating on the microcantilever backbone that will be excited by a solenoid such that applying a alternating current to it generates a magnetic field that interacts and excites the coated microcantilever. (b) iDrive, which is a technology that consists of triangular V-shaped cantilever that is gold coated. An alternating current is applied to the cantilever generating an electric field that will interact with a magnetic field generated by a permanent magnet. This is the so called Lorentz force excitation.

Choosing the optimal excitation method for the microcantilever is important for quantitative spectroscopy measurements. We put to test the 3 excitation schemes while in contact with a sample and determined the ideal for extracting quantitative information. Figure S1 shows the response spectra of a TR400PB cantilever acquired in contact with a glass slide in PBS for the 3 excitation methods. As shown clearly in Fig. S1, the excitation method that yields a smooth transfer function in which changes in amplitude and phase can be easily recorded and used to reconstruct the conservative and dissipative tip-sample interactions is the directly excited iDrive method. Thus, we chose

this method over the other 2 conventional actuation methods for our quantitative experiments.

b. Linearized spring and dashpot of a two-element Kelvin-Voigt viscoelasticity model

Because we use special soft cantilever with short tips for cell imaging, the hydrodynamic loading changes both the natural frequency and the damping of the cantilever as it comes closer to the sample surface [7-8]. As a consequence the theories for nanomechanical properties mapping requires two important considerations: (a) they must account for the difference (often significant) due to viscous hydrodynamics in the resonant response of the cantilever when located far and near the sample surface, and (b) the dynamics of the harmonically oscillating cantilevers interacting with the sample surface. From linear vibration theory, for a point mass oscillator of natural frequency ω_n and Q factor Q_n excited by a harmonic force:

$$\frac{\ddot{q}}{\omega_n^2}+q+\frac{1}{\omega_n Q_n}\dot{q}=\frac{F_{mag}\sin(\omega_{dr}t)}{k_{cant}}, \tag{S1}$$

the steady state vibration response is:

$$\begin{aligned}
&q(t)=A\sin(\omega_{dr}t-\phi),\\
&A=\frac{F_{mag}}{k_{cant}}\frac{1}{\sqrt{\left(1-\left(\frac{\omega_{dr}}{\omega_n}\right)^2\right)^2+\left(\frac{\omega_{dr}}{Q_n\omega_n}\right)^2}},\\
&\tan\phi=\frac{\left(\frac{\omega_{dr}}{Q_n\omega_n}\right)}{\left(1-\left(\frac{\omega_{dr}}{\omega_n}\right)^2\right)},\\
&\sin\phi=\frac{\left(\frac{\omega_{dr}}{Q_n\omega_n}\right)}{\sqrt{\left(1-\left(\frac{\omega_{dr}}{\omega_n}\right)^2\right)^2+\left(\frac{\omega_{dr}}{Q_n\omega_n}\right)^2}},\\
&\cos\phi=\frac{1-\left(\frac{\omega_{dr}}{\omega_n}\right)^2}{\sqrt{\left(1-\left(\frac{\omega_{dr}}{\omega_n}\right)^2\right)^2+\left(\frac{\omega_{dr}}{Q_n\omega_n}\right)^2}}.
\end{aligned} \tag{S2}$$

Using the above it can be easily shown that when the drive frequency is tuned to achieve maximum amplitude, the following hold:

$$\omega_{dr}\big/\omega_n = \sqrt{1-\tfrac{1}{2Q^2}},$$
$$A = \frac{F_{mag}}{k_{cant}}\frac{Q}{\sqrt{1-\tfrac{1}{4Q^2}}}, \qquad \text{(S3)}$$
$$\tan\phi = \sqrt{4Q^2-2}.$$

We will use these relationships several times in the following derivation.

Extracting local cellular mechanical properties:

Far from the sample, the natural frequency and Q-factor of the cantilever are ω_{far}, Q_{far}. The drive excitation frequency is tuned to achieve maximum amplitude. Hence, Eq. S2 applies and we have the following relationships for the amplitude and phase far from the sample A_{1far}, ϕ_{1far}

$$A_{1far} = \frac{1}{\sqrt{1-\tfrac{1}{4Q^2}}}\frac{F_{mag}Q_{far}}{k_{cant}},$$
$$\phi_{1far} = \tan^{-1}\sqrt{4Q_{far}{}^2-2}. \qquad \text{(S4)}$$

Note that when tuning the cantilever far from sample, the phase lag at the frequency of peak amplitude is not to be set to $\pi\big/2$ or 90° rather it should be set to $\tan^{-1}\sqrt{4Q_{far}{}^2-2}$ which say for $Q=2$ is a surprising 75° [9].

When brought near the sample and prior to the tip-sample interaction, the natural frequency and Q-factor of the cantilever change to ω_{near}, Q_{near} and as a consequence the amplitude and phase also change to A_{1near}, ϕ_{1near}. Thus the excitation frequency no longer corresponds to the drive frequency at which maximum amplitude occurs. So we invoke the more general Eq. S2:

$$A_{1near} = \frac{F_{mag}}{k_{cant}} \frac{1}{\sqrt{\left(1-(r)^2\right)^2 + \left(\frac{r}{Q_{near}}\right)^2}},$$

$$\sin\phi_{1near} = \frac{\left(\frac{r}{Q_{near}}\right)}{\sqrt{\left(1-r^2\right)^2 + \left(\frac{r}{Q_{near}}\right)^2}},$$

$$\cos\phi_{1near} = \frac{1-r^2}{\sqrt{\left(1-r^2\right)^2 + \left(\frac{r}{Q_{near}}\right)^2}}. \qquad \text{(S5)}$$

Where,

$$r = \frac{\omega_{dr}}{\omega_{near}}.$$

From Eqs. (S5a) and (S5c) we get:

$$1-\left(\frac{\omega_{dr}}{\omega_{near}}\right)^2 = \frac{F_{mag}\cos\phi_{1near}}{k_{cant}A_{1near}},$$

$$\frac{\omega_{dr}}{Q_{near}\omega_{near}} = \frac{F_{mag}\sin\phi_{1near}}{k_{cant}A_{1near}}.$$

Or, (S6)

$$\omega_{near}{}^2 = \frac{\omega_{dr}{}^2}{1-\frac{F_{mag}\cos\phi_{1near}}{k_{cant}A_{1near}}},$$

$$Q_{near} = \frac{F_{mag}\sin\phi_{1near}}{k_{cant}A_{1near}}\sqrt{1-\frac{F_{mag}\cos\phi_{1near}}{k_{cant}A_{1near}}}.$$

But from Eq. S5a,

$$F_{mag} = \frac{k_{cant}A_{1\,far}}{Q_{far}}\sqrt{1-\frac{1}{4Q^2}}, \qquad \text{(S6')}$$

which can be substituted in Eq. (5) to yield:

$$1-\left(\frac{\omega_{dr}}{\omega_{near}}\right)^2 = \cos\phi_{1near}\frac{A_{1far}}{A_{1near}}\frac{\sqrt{1-\frac{1}{4Q^2}}}{Q_{far}},$$

$$\frac{\omega_{dr}}{Q_{near}\omega_{near}} = \sin\phi_{1near}\frac{A_{1far}}{A_{1near}}\frac{\sqrt{1-\frac{1}{4Q^2}}}{Q_{far}}.$$

Or,

$$\omega_{near}{}^2 = \frac{\omega_{dr}{}^2}{1-\cos\phi_{1near}\frac{A_{1far}}{A_{1near}}\frac{\sqrt{1-\frac{1}{4Q^2}}}{Q_{far}}},$$

$$Q_{near} = \frac{\sqrt{1-\cos\phi_{1near}\frac{A_{1far}}{A_{1near}}\frac{\sqrt{1-\frac{1}{4Q^2}}}{Q_{far}}}}{\sin\phi_{1near}\frac{A_{1far}}{A_{1near}}\frac{\sqrt{1-\frac{1}{4Q^2}}}{Q_{far}}}. \tag{S7}$$

Dynamics while interacting with the sample

Now the equation of motion of the vibrating cantilever interacting with the soft cell becomes:

$$\frac{\ddot{q}}{\omega_{near}{}^2} + q + \frac{1}{\omega_{near}Q_{near}}\dot{q} = \frac{F_{mag}\sin(\omega_{dr}t) + F_{ts}}{k_{cant}}, \tag{S8}$$

whose steady state solution is expected in the form,

$$q(t) = A_0 + A_1\sin\left(\omega_{dr}t - \phi_1\right), \tag{S9}$$

so that the dynamic tip indentation into the sample is:

$$\begin{aligned}&\delta(t) = \delta_0 - A_1\sin\left(\omega_{dr}t - \phi_1\right),\\ &\textit{where}, \qquad .\\ &\delta_0 = -Z - A_0.\end{aligned} \tag{S10}$$

Accordingly,

$$\begin{aligned}F_{ts} &= F_{ts}(\delta_0) + k_{sample}^{dynamic}(\delta - \delta_0) + c_{sample}^{dynamic}\dot{\delta}\\ &= F_{ts}(\delta_0) - k_{sample}^{dynamic}A_1\sin(\omega_{dr}t - \phi_1) - c_{sample}^{dynamic}A_1\omega_{dr}\cos(\omega_{dr}t - \phi_1).\end{aligned} \tag{S11}$$

Substituting (S9) and (11) into (S8):

$$-\frac{\omega_{dr}^2 A_1 \sin\left(\omega_{dr}t-\phi_1\right)}{\omega_{near}{}^2}+A_0+A_1\sin\left(\omega_{dr}t-\phi_1\right)+\frac{A_1\omega_{dr}\cos(\omega_{dr}t-\phi_1)}{\omega_{near}Q_{near}}$$
$$=\frac{F_{mag}\sin(\omega_{dr}t-\phi_1+\phi_1)+F_{ts}(\delta_0)-k_{sample}^{dynamic}A_1\sin(\omega_{dr}t-\phi_1)-c_{sample}^{dynamic}A_1\omega_{dr}\cos(\omega_{dr}t-\phi_1)}{k_{cant}}, \tag{S12}$$

and collecting together terms in $\sin\left(\omega_{dr}t-\phi_1\right)$ or $\cos\left(\omega_{dr}t-\phi_1\right)$ and equating them on both sides of the equation (S12) gives us:

$$\begin{aligned} &k_{cant}A_0 = F_{ts}(\delta_0), \\ &k_{cant}A_1\left(1-\frac{\omega_{dr}^2}{\omega_{near}{}^2}\right) = F_{mag}\cos\phi_1 - k_{sample}^{dynamic}A_1, \\ &\frac{k_{cant}A_1\omega_{dr}}{\omega_{near}Q_{near}} = F_{mag}\sin\phi_1 - c_{sample}^{dynamic}A_1\omega_{dr}. \end{aligned} \tag{S13}$$

Eq. (S13) can be simplified using Eqs. (S7) and (S6') as thus:

$$\begin{aligned} &F_{ts}(\delta_0) = k_{cant}A_0, \\ &k_{sample}^{dynamic} = \left(\frac{k_{cant}A_{1far}}{Q_{far}A_1}\cos\phi_1 - \frac{k_{cant}A_{1far}}{Q_{far}A_{1near}}\cos\phi_{1near}\right)\sqrt{1-\frac{1}{4Q_{far}{}^2}}, \\ &c_{sample}^{dynamic} = \left(\frac{k_{cant}A_{1far}}{Q_{far}A_1\omega_{dr}}\sin\phi_1 - \frac{k_{cant}A_{1far}}{Q_{far}A_{1near}\omega_{dr}}\sin\phi_{1near}\right)\sqrt{1-\frac{1}{4Q_{far}{}^2}}. \end{aligned} \tag{S14}$$

These equations apply for both tapping mode observables and also for contact mode (with resonant excitation) observables.

c. Viscoelastic Sneddon's contact mechanics model

The fact that at each point on the image we can solve for the local force and damping gradients allows the extraction of unknown constitutive material properties, which are a more fundamental physical properties of cells, by using a tip-sample contact mechanic model of interest [9-10]. The Sneddon's contact mechanics model, which is a modification of the standard Hertz contact mechanics model for axisymmetric tips was used with a linear viscoelastic expansion [11]. The viscoelastic Sneddon's model for a cone-shaped AFM tip used in this study;

$$F_{ts} = \frac{2}{\pi}E^*\tan(\theta)\delta_0{}^2, \tag{S15}$$

with F_{ts}, tip-sample interaction force (N); $E^* = \frac{E^{storage}_{Sneddon-Cone}(\omega_{dr})}{\left(1-\upsilon_{sample}{}^2\right)} + i\frac{E^{loss}_{Sneddon-Cone}(\omega_{dr})}{\left(1-\upsilon_{sample}{}^2\right)}$, the complex effective sample modulus consisting of an elastic storage $E^{storage}_{Sneddon-Cone}$, and viscous loss $E^{loss}_{Sneddon-Cone}$ modulus representing the linear viscoelasticity of the sample evaluated at an average indentation depth (Pa); α, half-space cone angle of the cantilever; and δ_0, sample mean indentation [11-12].

Using the small oscillation assumption which means that the AFM probe oscillation amplitude is much smaller than the average indentation δ_0 on soft samples, the tip-sample interaction force as a Taylor series expression in $(\delta - \delta_0)$:

$$F_{ts} = F_{ts}(\delta_0) + k_{sample}(\delta - \delta_0) + c_{sample}\dot{\delta} + O(\varepsilon). \tag{S16}$$

Using Eq. S16 for small oscillations assumptions as above and neglecting the contribution of the higher order terms in F_{ts} Taylor series expansion and in the multiplicative correction, we find that:

$$\begin{aligned} k^{dynamic}_{sample} &= \frac{4}{\pi}\frac{E^{storage}_{Sneddon-Cone}}{\left(1-\nu^2_{sample}\right)}\tan(\alpha)\delta_0, \\ c^{dynamic}_{sample}\omega_{dr} &= \frac{4}{\pi}\frac{E^{loss}_{Sneddon-Cone}}{\left(1-\nu^2_{sample}\right)}\tan(\alpha)\delta_0. \end{aligned} \tag{S17}$$

Now, we present in further detail the method to quantify the local mechanical properties by combining the experimental multi-harmonic observables 0th and 1st data on live cells, let first write the expression of the dynamic and average tip indentation $\delta(t)$, δ_0 into the sample as:

$$\begin{aligned} \delta(t) &= -(Z+q) = -Z - A_0 - A_1\sin(\omega_{dr}t - \phi_1), \\ \delta_0 &= -Z + A_0. \end{aligned} \tag{S18}$$

where Z is the piezo movement, A_0 is the cantilever mean deflection, A_1 is the first harmonic amplitude, and ϕ_1 is the first harmonic phase lag.

Substituting Eqs. S18 into resulting Eqs. S16 and S17, we can solve for the unknown constitutive parameters:

$$\delta_0 = \sqrt{\frac{\pi}{2}\frac{\left(1-\nu_{sample}^2\right)}{E_{Sneddon-Cone}^{storage}\tan(\alpha)}F_{ts,CONS}^0},$$

$$E_{Sneddon-Cone}^{storage} = \left(\sqrt{\frac{\pi}{8}}\frac{F_{ts,CONS}^1}{A_1\sqrt{F_{ts,CONS}^0}}\right)^2\frac{\left(1-\nu_{sample}^2\right)}{\tan(\alpha)},$$

$$E_{Sneddon-Cone}^{loss} = \left(\sqrt{\frac{\pi}{8}}\frac{F_{ts,DISS}^1}{A_1\sqrt{F_{ts,CONS}^0}}\right)^2\frac{\left(1-\nu_{sample}^2\right)}{\tan(\alpha)}.$$

(S19)

Finally, the force harmonics (0th, and 1st Fourier components of the tip-sample interaction force for live cells) of the tip-sample interaction force in terms of the multi-harmonic observables (A_0, A_1, and ϕ_1) were previously derived in (Raman *et al.* [10]) and with a slight modification using the hydrodynamic correction derived in (Cartagena *et al.* [9]) for soft and low Q factor microcantilevers tuned to the peak amplitude of the resonance curve far from the surface, the resulting formulae are:

$$\begin{aligned} F_{ts,CONS}^0 &= k_{cant}A_0, \\ F_{ts,CONS}^1 &= \frac{k_{cant}A_{1far}}{Q_{far}}\left(-\cos(\phi_1)+\frac{A_1}{A_{1near}}\cos(\phi_{1near})\right)\sqrt{1-\frac{1}{4Q_{far}^2}}, \\ F_{ts,DISS}^1 &= \frac{k_{cant}A_{1far}}{Q_{far}}\left(-\sin(\phi_1)+\frac{A_1}{A_{1near}}\sin(\phi_{1near})\right)\sqrt{1-\frac{1}{4Q_{far}^2}}. \end{aligned} \qquad \text{(S20)}$$

d. Bottom effect cone correction (BECC) of the Sneddon's model with viscoelasticity extension

Sneddon's contact mechanics model requires small sample indentations <10% of sample height. However, a model that takes into account the artifact generated by moderate and large indentations of conical tips in AFM measurements on thin samples and adherent cells is required. In this case, we chose to use the BECC contact model [13], which is a multiplicative analytical correction done to the commonly used Sneddon's model. This is a non-artifactual contact mechanics model that takes into consideration topographical effects by large indentations in soft samples like live cells. For a cell thickness of ~4 µm as used in this work, an indentation of larger than 400-800 nm would be needed to violate the assumptions of the standard Sneddon's model. However all the measurements made here have been for indentations less than 400 nm. Because as shown before [9-10] cantilever oscillation amplitude is small compared to indentations, it is reasonable to use a linear viscoelastic model to extract the

constitutive material properties like elastic storage and viscous loss modulus. Thus, the resulting tip-sample interaction force model is:

$$F_{ts} = \frac{8E^* \tan(\alpha)\delta^2}{3\pi}\left(1+1.7795\frac{2\tan(\alpha)\delta}{\pi^2 h}+16(1.7795)^2\tan^2(\alpha)\frac{\delta^2}{h^2}+O\left(\frac{\delta^3}{h^3}\right)\right), when \delta > 0,$$
$$= 0, otherwise,$$

(S21)

where $E^* = \frac{E_{BECC}^{storage}(\omega_{dr})}{\left(1-\upsilon_{sample}{}^2\right)} + i\frac{E_{BECC}^{loss}(\omega_{dr})}{\left(1-\upsilon_{sample}{}^2\right)}$ is the complex effective sample modulus consisting of an elastic storage $E_{BECC}^{storage}$, and viscous loss E_{BECC}^{loss} modulus representing the linear viscoelasticity of the sample evaluated at an average indentation depth. δ, h, and α, respectively, are the indentation, the height of the sample at that location, and the half-opening angle of the cone.

Using Eq. S16 for small oscillations assumptions as previously presented and neglecting the contribution of the higher order terms in F_{ts} Taylor series expansion and in the multiplicative correction, we find that:

$$k_{sample}^{dynamic} = \frac{8E_{BECC}^{storage}\tan(\alpha)\delta_0}{3\pi\left(1-\nu_{sample}^2\right)}\left(2+3(1.7795)\frac{2\tan(\alpha)\delta_0}{\pi^2 h}+64(1.7795)^2\tan^2(\alpha)\frac{\delta_0^2}{h^2}\right),$$
$$c_{sample}^{dynamic}\omega_{dr} = \frac{8E_{BECC}^{loss}\tan(\alpha)\delta_0}{3\pi\left(1-\nu_{sample}^2\right)}\left(2+3(1.7795)\frac{2\tan(\alpha)\delta_0}{\pi^2 h}+64(1.7795)^2\tan^2(\alpha)\frac{\delta_0^2}{h^2}\right). \quad \text{(S22)}$$

Substituting Eqs. S18 into resulting Eqs. S16 and S22, and evaluating the Fourier coefficients of the tip-sample interaction force are:

$$F_{ts,CONS}^0 = \frac{8E_{BECC}^{storage}\tan(\alpha)\delta_0^2}{3\pi\left(1-\nu_{sample}^2\right)}\left(1+1.7795\frac{2\tan(\alpha)\delta_0}{\pi^2 h}+16(1.7795)^2\tan^2(\alpha)\frac{\delta_0^2}{h^2}\right),$$
$$F_{ts,CONS}^1 = \frac{-8E_{BECC}^{storage}\tan(\alpha)\delta_0}{3\pi\left(1-\nu_{sample}^2\right)}\left(2+3(1.7795)\frac{2\tan(\alpha)\delta_0}{\pi^2 h}+64(1.7795)^2\tan^2(\alpha)\frac{\delta_0^2}{h^2}\right)A_1,$$
$$F_{ts,DISS}^1 = \frac{-8E_{BECC}^{loss}\tan(\alpha)\delta_0}{3\pi\left(1-\nu_{sample}^2\right)}\left(2+3(1.7795)\frac{2\tan(\alpha)\delta_0}{\pi^2 h}+64(1.7795)^2\tan^2(\alpha)\frac{\delta_0^2}{h^2}\right)A_1.$$

(S23)

where $F_{ts,CONS}^{0}$ is the 0th Fourier coefficient of the conservative interaction force, $F_{ts,CONS}^{1}$ is the 1st Fourier coefficient of the conservative interaction force, and $F_{ts,DISS}^{1}$ is the 1st Fourier coefficient of the dissipative interaction force (Eq. S20).

Defining dimensionless parameter $\delta_h = \dfrac{\delta_0}{h}$ (average indentation against topography) and rearranging the equations:

$$\frac{F_{ts,CONS}^{0} A_1}{F_{ts,CONS}^{1} h} = \frac{-(\delta_h)\left(1+1.7795\dfrac{2\tan(\alpha)\delta_h}{\pi^2}+16(1.7795)^2\tan^2(\alpha)\delta_h^2\right)}{\left(2+3(1.7795)\dfrac{2\tan(\alpha)\delta_h}{\pi^2}+64(1.7795)^2\tan^2(\alpha)\delta_h^2\right)},$$

$$\frac{F_{ts,CONS}^{0}}{h^2} = \frac{8E_{BECC}^{storage}\tan(\alpha)\delta_h^2}{3\pi\left(1-\nu_{sample}^2\right)}\left(1+1.7795\frac{2\tan(\alpha)\delta_h}{\pi^2}+16(1.7795)^2\tan^2(\alpha)\delta_h^2\right), \qquad \text{(S24)}$$

$$\frac{F_{ts,CONS}^{1}}{hA_1} = \frac{-8E_{BECC}^{storage}\tan(\alpha)\delta_h}{3\pi\left(1-\nu_{sample}^2\right)}\left(2+3(1.7795)\frac{2\tan(\alpha)\delta_h}{\pi^2}+64(1.7795)^2\tan^2(\alpha)\delta_h^2\right),$$

$$\frac{F_{ts,DISS}^{1}}{hA_1} = \frac{-8E_{BECC}^{loss}\tan(\alpha)\delta_h}{3\pi\left(1-\nu_{sample}^2\right)}\left(2+3(1.7795)\frac{2\tan(\alpha)\delta_h}{\pi^2}+64(1.7795)^2\tan^2(\alpha)\delta_h^2\right).$$

These expressions clearly link the experimental observables to quantitatively extract the nanoscale constitutive mechanical properties $E_{BECC}^{storage}$ and E_{BECC}^{loss}. A MATLAB code has been written that performs a nonlinear least squares best fit of those unknown nanomechanical properties δ_0, $E_{BECC}^{storage}$ and E_{BECC}^{loss} that best match the measured force harmonics, formulas Eqs. S24 and S20 [9-10]. It is important to keep in mind that these equations actually extract the effective properties of the live cell at a specific mean indentation δ_0 and excitation frequency ω_{dr} [9]. With the above briefly discussed theory and the maps of multi-harmonic amplitudes and phases (A_0, A_1, and ϕ_1), that can be easily acquired on a live cancer cell *in vitro*, it's possible to map the mean indentation (δ_0) and the complex elastic modulus of the viscoelastic sample ($E_{BECC}^{storage}$, and E_{BECC}^{loss}).

e. Bimodal AFM imaging for viscoelastic property mapping

For bimodal experiments, because of the cell softness and the low Q-factor of the soft cantilever in liquids, the vibrational mode shapes of the cantilever are assumed to be unperturbed. Moreover, since the cantilever oscillations are much smaller than the net indentation into the cell it can be assumed that the equation-of-motion of the cantilever can be separated into two independent simple harmonic oscillators [14]. Therefore, we

can model the governing dynamics of the soft cantilever in permanent contact on the cell surface in liquid as:

$$\frac{\ddot{q}_1}{\omega_1^{\ 2}}+\frac{\dot{q}_1}{\omega_1 Q_1}+q_1=\frac{F_{mag}\sin(\omega_{dr}t)+F_{ts}}{k_{cant,1}},$$
$$\frac{\ddot{q}_2}{\omega_2^{\ 2}}+\frac{\dot{q}_2}{\omega_2 Q_2}+q_2=\frac{F_{mag}\sin(\omega_{dr}t)+F_{ts}}{k_{cant,2}}, \qquad \text{(S25)}$$

where q_1 is the contribution to tip deflection of 1st eigenmode, q_2 is the contribution to tip deflection of 2nd eigenmode, ω_1 is the resonance frequency of 1st eigenmode, ω_2 is the resonance frequency of 2nd eigenmode, Q_1 is the quality factor of 1st eigenmode, Q_2 is quality factor of 2nd eigenmode, $k_{cant,1}$ is the effective spring constant of 1st eigenmode, and $k_{cant,2}$ is the effective spring constant of 2nd eigenmode, respectively. The constitutive unknown parameters are solved by combining the reconstructed tip-sample interaction force and the Sneddon's contact mechanics model. Following the derivation of Supplementary Information Section c, the resulting analytical equations to solve for the unknown constitutive parameters stiffness $k_{sample,1}^{dynamic}$, $k_{sample,2}^{dynamic}$ and $c_{sample,1}^{dynamic}$, $c_{sample,2}^{dynamic}$ are:

$$\delta_{0,1} = \sqrt{\frac{\pi}{2} \frac{\left(1-\nu_{sample}^2\right)}{E_{Sneddon-Cone}^{storage,1} \tan(\alpha)} F_{ts1,CONS}^{0}},$$

$$E_{Sneddon-Cone}^{storage,1} = \left(\sqrt{\frac{\pi}{8}} \frac{F_{ts1,CONS}^{1}}{A_1 \sqrt{F_{ts1,CONS}^{0}}} \right)^2 \frac{\left(1-\nu_{sample}^2\right)}{\tan(\alpha)},$$

$$E_{Sneddon-Cone}^{loss,1} = \left(\sqrt{\frac{\pi}{8}} \frac{F_{ts1,DISS}^{1}}{A_1 \sqrt{F_{ts1,CONS}^{0}}} \right)^2 \frac{\left(1-\nu_{sample}^2\right)}{\tan(\alpha)},$$

and,

$$\delta_{0,2} = \sqrt{\frac{\pi}{2} \frac{\left(1-\nu_{sample}^2\right)}{E_{Sneddon-Cone}^{storage,2} \tan(\alpha)} F_{ts2,CONS}^{0}},$$

$$E_{Sneddon-Cone}^{storage,2} = \left(\sqrt{\frac{\pi}{8}} \frac{F_{ts2,CONS}^{1}}{A_1 \sqrt{F_{ts2,CONS}^{0}}} \right)^2 \frac{\left(1-\nu_{sample}^2\right)}{\tan(\alpha)},$$

$$E_{Sneddon-Cone}^{loss,2} = \left(\sqrt{\frac{\pi}{8}} \frac{F_{ts2,DISS}^{1}}{A_1 \sqrt{F_{ts2,CONS}^{0}}} \right)^2 \frac{\left(1-\nu_{sample}^2\right)}{\tan(\alpha)}. \qquad \text{(S26)}$$

f. Additional images of nanomechanical properties of rat fibroblast cells

In this work we performed multiple fast AFM imaging of live rat fibroblasts and MDA-MB-231 human breast cancer cells using Lorentz-force microcantilever excitation with feedback on the cantilever mean deflection. After imaging we extracted their nanomechanical properties. In Fig S3 we provide additional images of a living fibroblast cell in culture media, showing that this novel technique can be easily implemented with repeatability and confidence yielding reasonable quantitative nanomechanical values.

Figure S4 shows the viscoelastic tangent loss $\tan\delta$ maps obtained at two widely spaced high frequencies (7 kHz and 61 kHz) on a live rat fibroblast cell in culture media. $\tan\delta$ maps, Figs. S4(a and b), clearly shows the classical viscoelastic frequency dependence. Figure S4c is the difference between the low and high frequency $\tan\delta$ maps showing a reduction by ~0.3-0.9 on the cell.

g. Adhesion experiments

The expression in MDA-MB-231 cells of Syk decreases cell motility and enhances adhesion. To confirm that this effect is an intrinsic property of the active kinase, we compared cells either lacking Syk or expressing Syk-EGFP (wild-type Syk with a green fluorescent protein tag) or Syk-AQL-EGFP, an analog-sensitive version of Syk. The treatment with 1-NM-PP1, an orthogonal inhibitor of Syk-AQL-EGFP, of cells expressing

the engineered kinase, but not the wild-type enzyme, reduced adhesion to the level seen with Syk-deficient cells (Fig. S5). These experiments illustrate the ability of Syk to enhance cell adhesion in a manner dependent on its catalytic activity.

h. How well the BECC contact mechanics model fits the experimental data

We used a nonlinear least-squared fit algorithm to best fit the unknown physical properties to the experimental data dynamic AFM observables. In order to check the applicability of the contact model and the experimental data we extracted the residuals and resnorm of the fit. The residuals measure the differences between a data point and the corresponding mechanics models estimate, therefore the smaller the difference the better the fit. However, residuals can be positive or negative making it difficult sometimes to judge if the fit is good. The resnorm is a better estimate consisting in the sum of squared residuals. Figure S6 show the extracted values for the residuals and resnorm are very small confirming the goodness of the fit.

i. Movies of additional MDA-MB-231 human breast cancer cell expressing Syk and treated with inhibitor

We present an additional example of the MDA-MB-231 human breast cancer cells expressing Syk-AQL-EGFP after addition of 1-NMPP1 for Syk inhibition. Movies S1-S3 show the time-varying changes in the multi-harmonic observables signals. Movies S4-S6 show the extracted nanomechanical properties presenting progressive changes in the elastic $E_{sample}^{storage}$ and viscous E_{sample}^{loss} and the indentation δ_0. The movies have a total of 14 images. Each image was obtained at 1 min 30 s intervals, with a total time of 21 mins.

The movies provide insights into the kinetics of cytoskeletal changes. Interestingly, rapid changes in the cytoskeletal architecture at the cell periphery could be visualized within 1.5 min including the formation and movement of lateral actin bands or transverse arcs characteristic of retrograde actin flow that preceded the release of focal adhesions. Thus, the rapid loss of Syk activity was correlated with dramatic rearrangements in the cortical actin cytoskeleton.

References

1. Xu, X. & Raman, A. Comparative dynamics of magnetically, acoustically, and Brownian motion driven microcantilevers in liquids. *J. Appl. Phys.* **102**, 034303 (2007).
2. Enders, O., Korte, F. & Kolb H. A. Lorentz-force-induced excitation of cantilevers for oscillation-mode scanning probe microscopy. *Surf. Interface Anal.* **36**,119-123 (2004).
3. Kiracofe, D., Kobayashi, K., Labuda, A., Raman, A. & Yamada, H. High efficiency laser photothermal excitation of microcantilever vibrations in air and liquids. *Rev. Sci. Instrum.* **82**, 103702 (2011).
4. Labuda, A. *et al.* Comparison of photothermal and piezoacoustic excitation methods for frequency and phase modulation atomic force microscopy in liquid environments. *AIP Advances* **1**, 022136 (2011).
5. Rabe, U. & Arnold, W. Acoustic microscopy by atomic force microscopy. *Appl. Phys. Lett.* **64**, 1493 (1994).
6. Kiracofe, D. & Raman, A. Quantitative force and dissipation measurements in liquids using piezo-excited atomic force microscopy: a unifying theory. *Nanotechnology* **22**, 485502 (2011).
7. Tung, R. C., Jana, A. & Raman, A. Hydrodynamic loading of microcantilevers oscillating near rigid walls. *J. Appl. Phys.* **104**, 114905 (2008).
8. Xu, X., Carrasco, C., de Pablo, P. J., Gomez-Herrero, J. & Raman, A. Unmasking imaging forces on soft biological samples in liquids when using dynamic atomic force microscopy: a case study on viral capsids. *Biophys. J.* **95**, 2520-2528 (2008).
9. Cartagena, A. & Raman, A. Local viscoelastic properties of live cells investigated using dynamic and quasi-static atomic force microscopy methods. *Biophys. J.* **5**, 3575-3585 (2014).
10. Raman, A. *et al.* Mapping nanomechanical properties of live cells using multi-harmonic atomic force microscopy. *Nature Nanotech.* **6**, 809-814 (2011).
11. Sneddon, I.N. (1965) The relation between load and penetration in the axisymmetric boussinesq problem for a punch of arbitrary profile. *Int. J. Engng. Sci. 3*, 47-57.
12. Alcaraz, J., Buscemi, L., Grabulosa, M., Trepat, X., Fabry, B., Farré, R., and Navajas, D. (2003) Microrheology of human lung epithelial cells measured by atomic force microscopy. *Biophys. J. 84*, 2071-2079.
13. Gavara, N. & Chadwick, R. S. Determination of the elastic moduli of thin samples and adherent cells using conical atomic force microscope tips. *Nature Nanotech*. **7**, 733-736 (2012).
14. Xu, X., Melcher, J. & Raman, A. Accurate force spectroscopy in tapping mode atomic force microscopy in liquids. *Phys. Rev. B* **81**, 035407 (2010).

Supplementary Figures

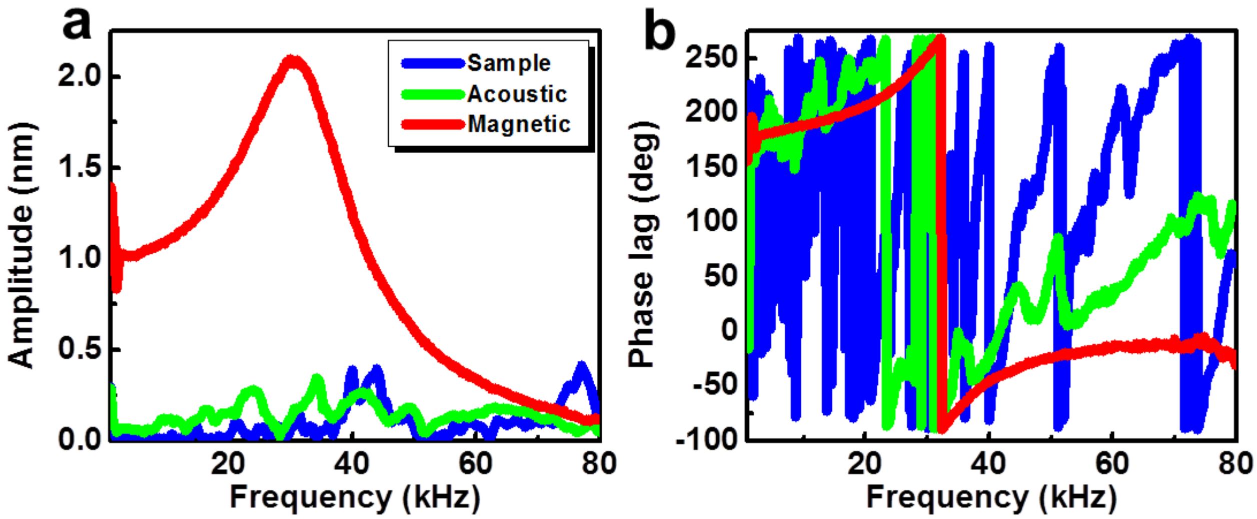

Figure S1. Using different excitation techniques for tip-surface coupled AFM probes in liquids. Tune curves (**a**) amplitude (nm) and (**b**) phase lag (deg) performed using different cantilever excitation methods on a glass surface: acoustic (red), sample (green), and iDrive-magnetic (blue). IDrive is the only excitation method that retains the transfer function of a single harmonic oscillator. This clearly shows that magnetic excitation is the natural choice for quantitative measurements in liquids.

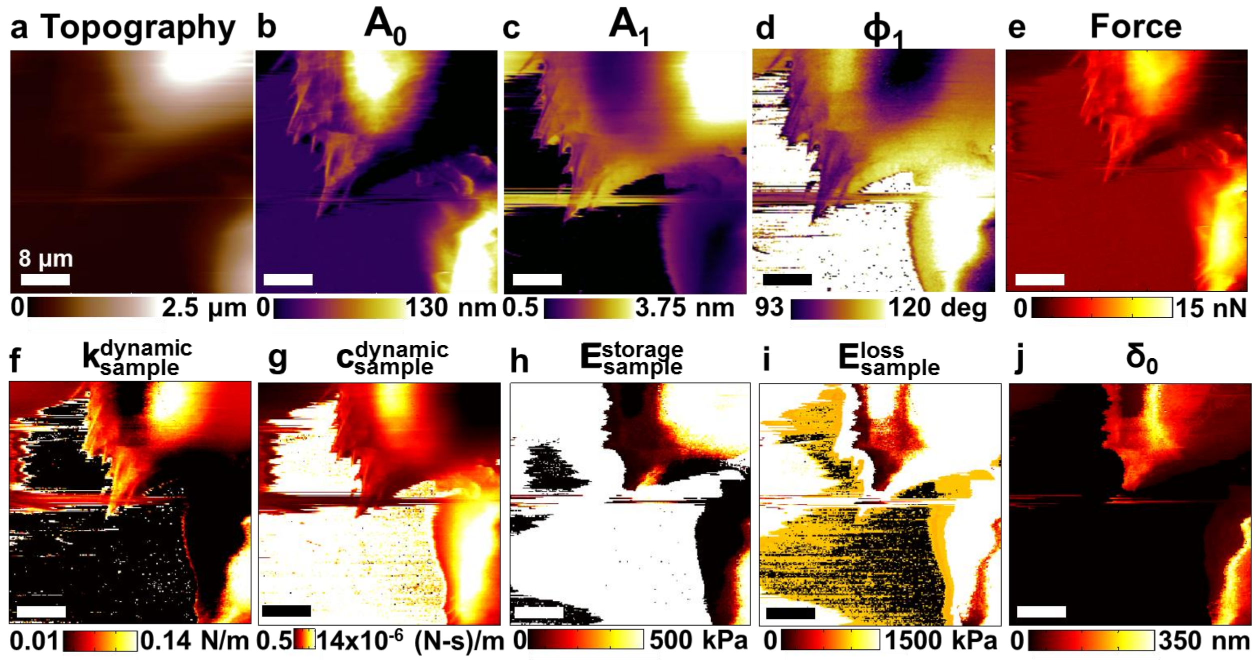

Figure S2. Additional nanomechanical images of live rat fibroblast cells. (**a**) Topography image of a live rat fibroblast cell scanned in physiological media solution using Lorentz excited cantilever with A_0 regulation (see Materials and Methods). (**b-d**) Multi-harmonic images of (A_0, A_1, ϕ_1) acquired simultaneously with topography showing high resolution subcellular contrast related to the local physical properties. (**f-g**) Maps of local dynamic stiffness $k_{sample}^{dynamic}$ and damping $c_{sample}^{dynamic}$ extracted from the multi-harmonic data and using the linear model described in the Supplementary Information. (**h-j**) Maps of local storage modulus $E_{sample}^{storage}$, local loss modulus E_{sample}^{loss}, and mean indentation δ_0 extracted using the multi-harmonic data and the BECC contact mechanics model described in the Supplementary Information B. Imaging parameters; f_1=7.9 kHz, k_{cant}=87.41 pN/nm, Q=1.75, α=35°, and A_{0sp}=15 nm. The scale bar on images represents 8 µm (size; 40x40 µm^2, pixels; 256x256, acquisition time; 3 min 30s).

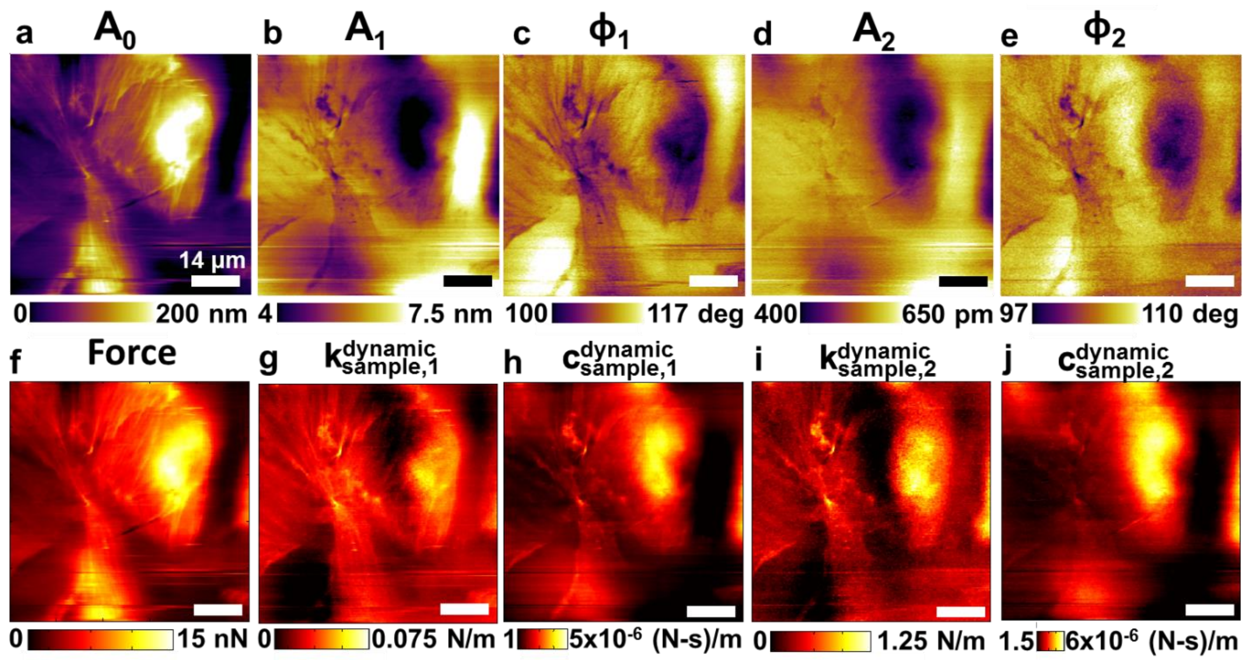

Figure S3. Additional rat fibroblast cell using new technique with bimodal. Multi-frequency observables images (**a**) DC signal mean deflection A_0 , first and second flexural eigenmodes amplitudes and phases (**b-c**) A_1 , ϕ_1 , and (**d-e**) A_2 , ϕ_2 obtained simultaneously using the previously described dynamic AFM method. (**f-g**) Maps of local dynamic stiffness $k_{sample,1}^{dynamic}$ (N m^{-1}) and damping $c_{sample,1}^{dynamic}$ (N s m^{-1}) extracted from the measured first mode data (f_1=7.06 kHz) using the theory described in the text and Supplementary Information. (**h-i**) Maps of local dynamic stiffness $k_{sample,2}^{dynamic}$ (N m^{-1}) and damping $c_{sample,2}^{dynamic}$ (N s m^{-1}) extracted from the measured second mode data (f_2 =61.33 kHz) using the theory described in the text and Supplementary Information. This shows multi-frequency can be combined with this technique enabling additional compositional contrast channels revealing unrelated subcellular features. Topography and multi-modal observables were not taken simultaneously. Imaging parameters; $k_{cant,1}$=77.19 pN/nm, Q_1=1.7, $k_{cant,2}$=1.33 N/m, Q_2 =3, and A_{0sp} =36 nm. The scale bar on images represents 14 µm (size; 70x70 µm^2, pixels; 256x256, acquisition time; 2 mins).

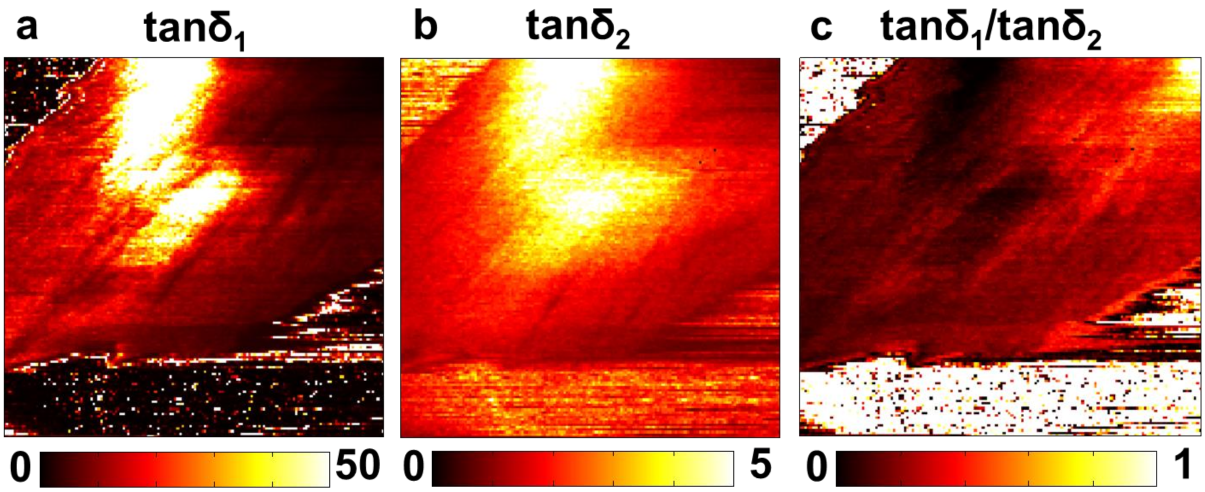

Figure S4. Viscoelastic loss tangent maps at low and high frequencies. (**a**) tanδ map at low frequency 7 kHz and (**b**) tanδ map at high frequency 61 kHz acquired for an adherent live fibroblast cell in culture media. (**c**) Reduction in viscoelastic loss tangent is observed by ~0.3-0.9.

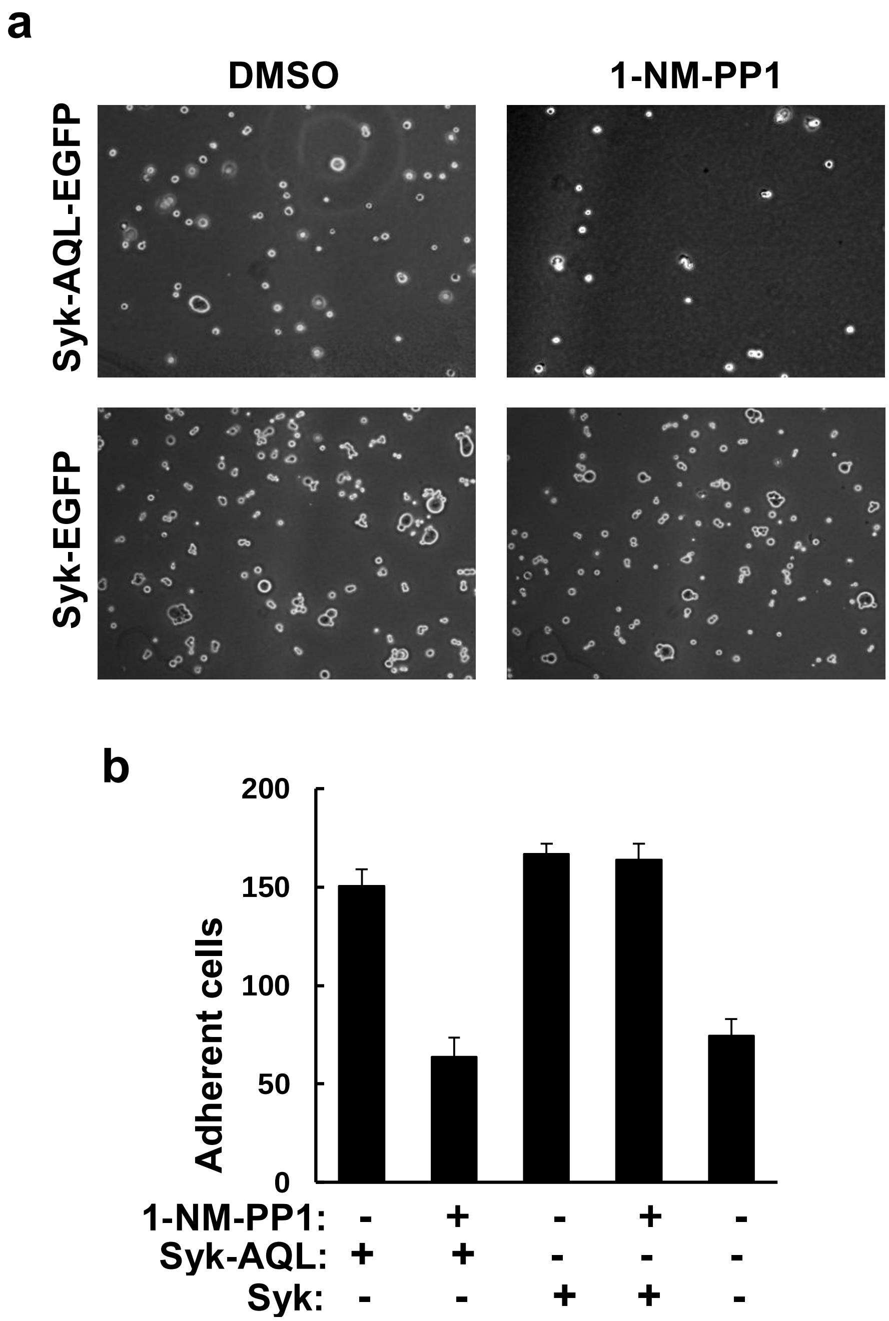

Figure S5. Effect of Syk inhibition on cell adhesion. MDA-MB-231 cells lacking Syk, or expressing either Syk-EGFP or Syk-AQL-EGFP (5 X 10^5) were treated with 1-NM-PP1 (5 μM) or DMSO carrier alone and plated in a 6-well culture plate for 30 min. Wells were washed three-times with PBS and adherent cells visualized by light microscopy and counted. Examples of typical fields of MDA-MB-231 cells expressing Syk-EGFP or Syk-AQL-EGFP are illustrated in panel (**a**). An analysis of adherent cell counts from three separate experiments, each performed in triplicate are shown in panel (**b**).

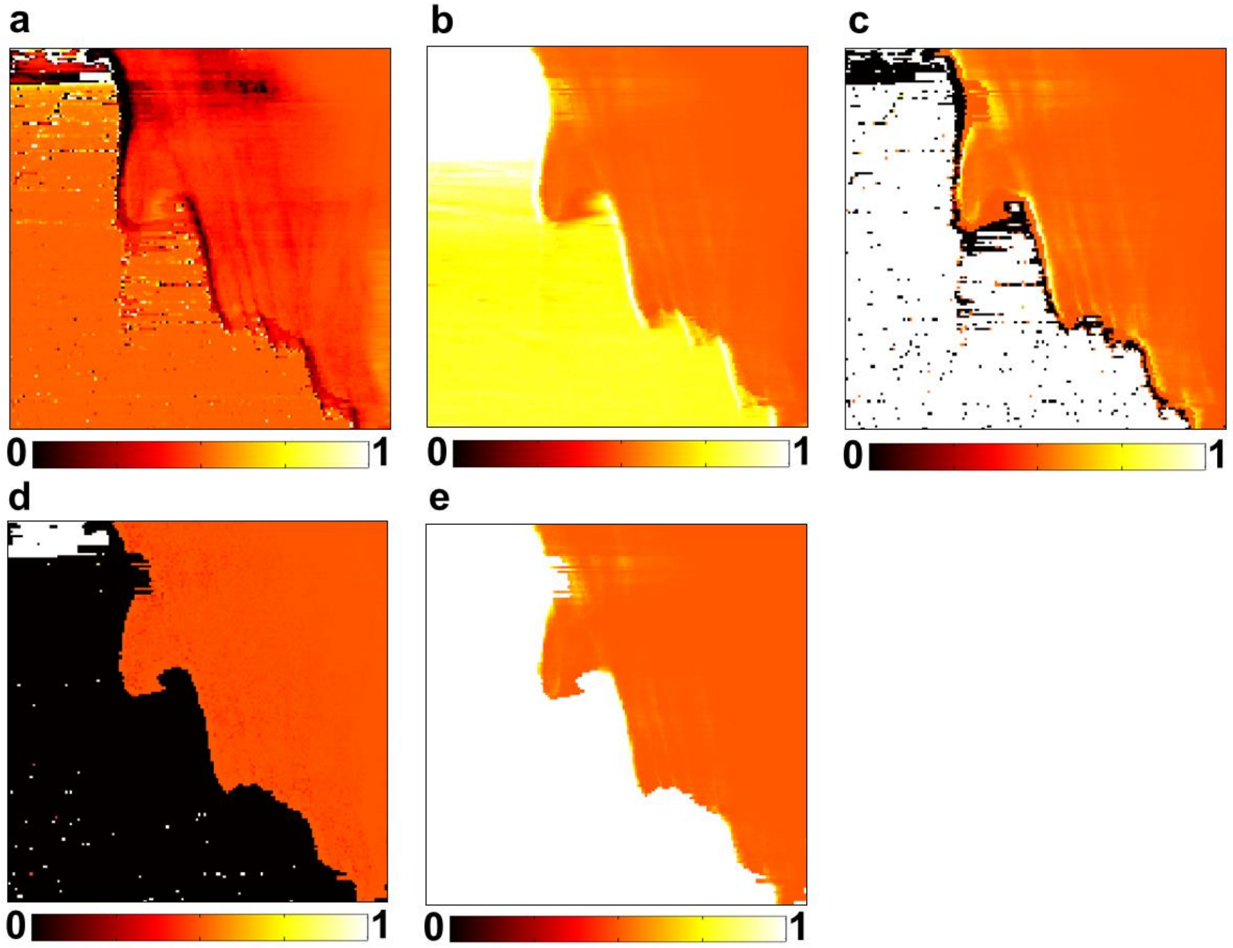

Figure S6. BECC contact mechanics model fits well to the experimental data for nanomechanical properties extraction. (a-d) The residual maps showing the relationship between the experimental data and the estimated parameters. Residuals are found to be very low for all cases (~0.1). **(e)** The resnorm of the residuals is indeed small suggesting the fit is good.

Supplementary Movies

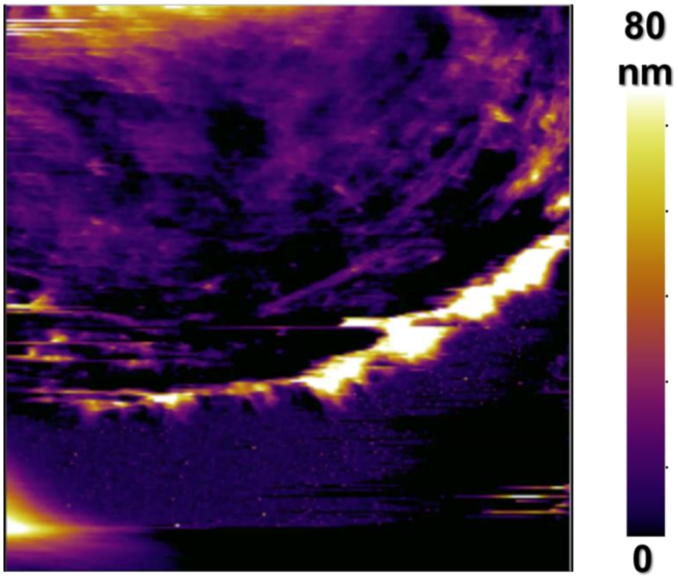

Movie S1. Progressive variation of cantilever mean deflection (A_0) signal showing visualization of cytoskeleton cortical actin network on a human breast cancer cell.

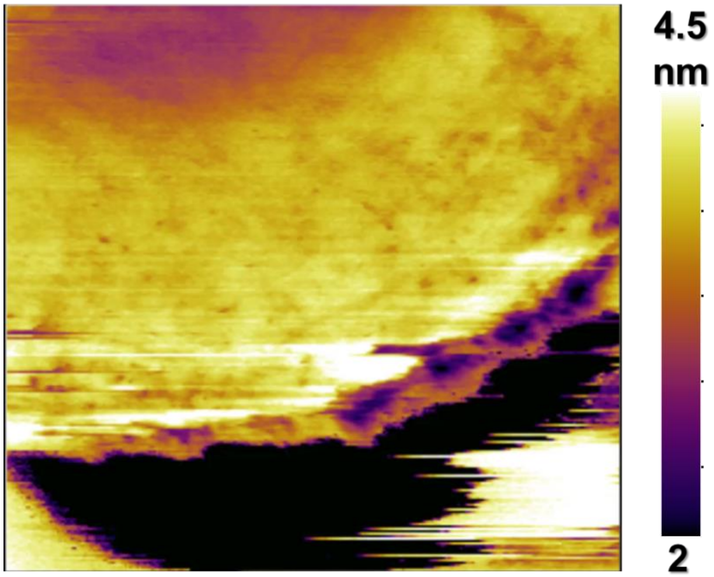

Movie S2. Progressive variation of first harmonic oscillation amplitude (A_1) signal on a human breast cancer cell.

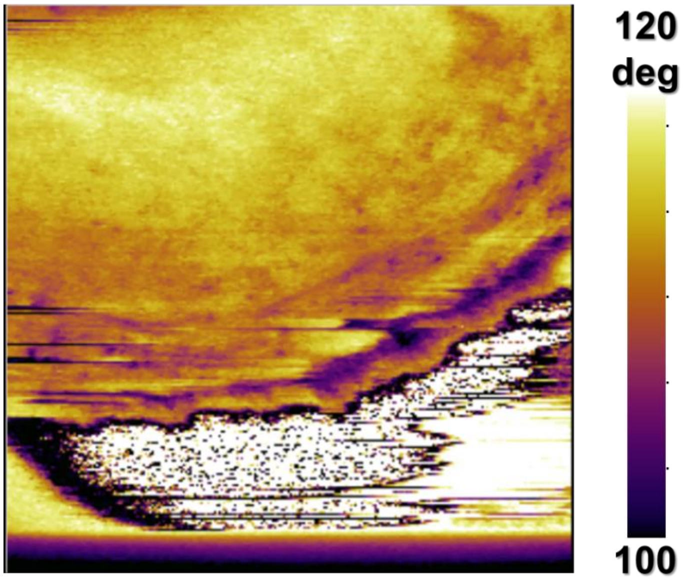

Movie S3. Progressive variation of first harmonic phase lag (ϕ_1) signal on a human breast cancer cell.

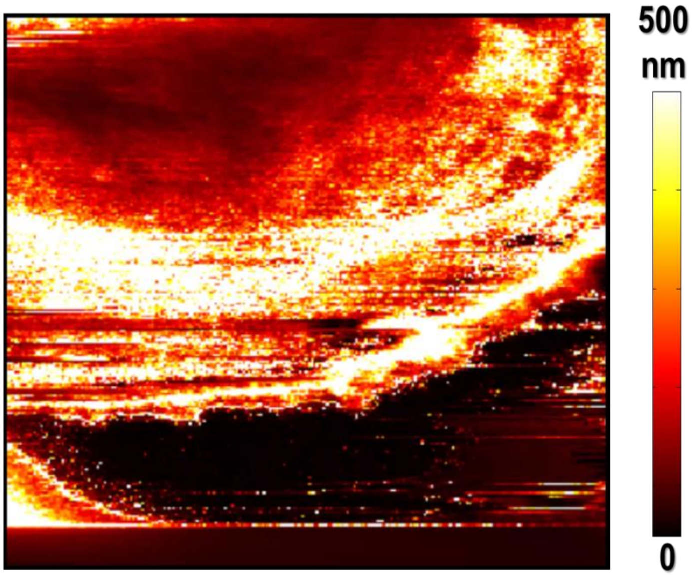

Movie S4. Progressive variation of nanoscale mean indentation (δ_0) map on a human breast cancer cell.

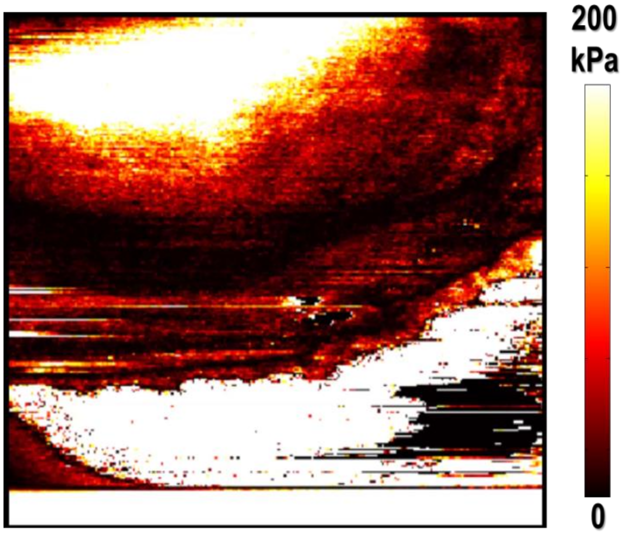

Movie S5. Progressive variation of nanoscale elastic storage ($E^{storage}_{Sneddon-Cone}$) modulus on a human breast cancer cell.

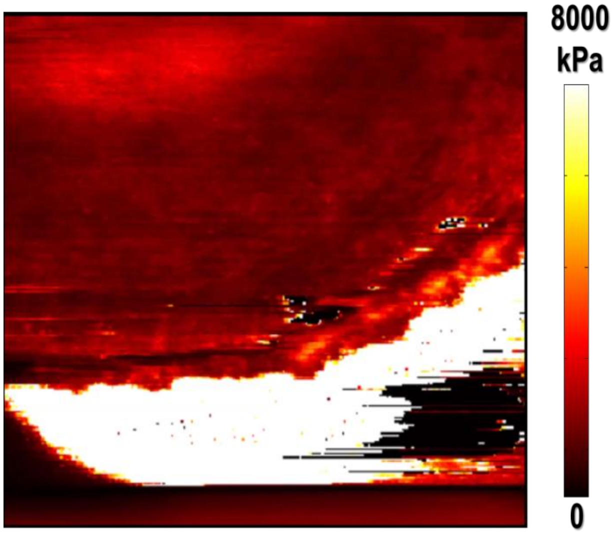

Movie S6. Progressive variation of nanoscale viscous loss ($E^{loss}_{Sneddon-Cone}$) modulus on a human breast cancer cell.
